# Prediction of plant-derived xenomiRs from plant miRNA sequences using random forest and one-dimensional convolutional neural network models

**DOI:** 10.1186/s12864-018-5227-3

**Published:** 2018-11-26

**Authors:** Qi Zhao, Qian Mao, Zheng Zhao, Tongyi Dou, Zhiguo Wang, Xiaoyu Cui, Yuanning Liu, Xiaoya Fan

**Affiliations:** 1 Sino-Dutch Biomedical and Information Engineering School, Northeastern University, Shenyang, 110169 Liaoning China; 2 Light Industry College, Liaoning University, Shenyang, 110036 Liaoning China; 3 Department of Network Engineering, Zhengzhou Science and Technology Institute, Zhengzhou, 450000 Henan China; 4 School of Life Science and Medicine, Dalian University of Technology, Panjin, 124221 China; 5 Department of Nuclear Medicine, The General Hospital of Shenyang Military Area Command, Shenyang, 110840 China; 6 Computer Science and Technology College, Jilin University, Changchun, 130012 China; 7 Bio-, Electro- And Mechanical Systems, Université Libre de Bruxelles, Avenue F.D. Roosevelt 50 CP165/56, 1050 Brussels, Belgium

**Keywords:** miRNA, Plant-derived xenomiR, Cross-kingdom regulation, Selective absorption, Statistics analysis, Machine learning

## Abstract

**Background:**

An increasing number of studies reported that exogenous miRNAs (xenomiRs) can be detected in animal bodies, however, some others reported negative results. Some attributed this divergence to the selective absorption of plant-derived xenomiRs by animals.

**Results:**

Here, we analyzed 166 plant-derived xenomiRs reported in our previous study and 942 non-xenomiRs extracted from miRNA expression profiles of four species of commonly consumed plants. Employing statistics analysis and cluster analysis, our study revealed the potential sequence specificity of plant-derived xenomiRs. Furthermore, a random forest model and a one-dimensional convolutional neural network model were trained using miRNA sequence features and raw miRNA sequences respectively and then employed to predict unlabeled plant miRNAs in miRBase. A total of 241 possible plant-derived xenomiRs were predicted by both models. Finally, the potential functions of these possible plant-derived xenomiRs along with our previously reported ones in human body were analyzed.

**Conclusions:**

Our study, for the first time, presents the systematic plant-derived xenomiR sequences analysis and provides evidence for selective absorption of plant miRNA by human body, which could facilitate the future investigation about the mechanisms underlying the transference of plant-derived xenomiR.

**Electronic supplementary material:**

The online version of this article (10.1186/s12864-018-5227-3) contains supplementary material, which is available to authorized users.

## Background

miRNAs and their gene expression regulation function in eukaryotes is one of the most important discoveries in recent years [1]. It has been well established that endogenous miRNAs could degrade or silence mRNAs to mediate gene expression by binding RNA-induced silencing complex (RICS) in a sequence-specific manner [2]. Meanwhile, although still controversial, new hypotheses about extracellular miRNA have been continually proposed, e.g., exosomal miRNA [3, 4], circulating miRNA [5, 6] and exogenous miRNA (xenomiR) [7, 8].

Due to the possibility of cross-kingdom regulation, plant-derived xenomiR hypothesis has received great attention since first proposed in 2012 [7]. Plant-derived xenomiRs were defined as the miRNAs derived from plants which are capable of transferring into human or animal bodies. Subsequently, plant-derived xenomiRs have been detected in different tissues or body fluids of several species of animals, including human [7], mice [7], pig [9], panda [10] and silkworm [11]. And their relevance to many diseases, such as cardiovascular diseases [7, 12], tumor [13, 14], chronic-inflammation [15], influenza [16], benign prostatic hyperplasia [17] and pulmonary fibrosis [18], were also proposed. However, many mechanisms of plant-derived xenomiRs in keeping stable in gastrointestinal (GI) track, transferring across GI track, entering cells or being secreted by cells are still unknown. Of note, negative results have also been reported in some biological experiments and computational analyses, making this issue very contentious. Some biological experiments-based studies claimed the plant-derived miRNAs detected in animal samples were contamination during experiments instead of bona fide xenomiRs [19–24]. Of interest, exogenous non-diet derived miRNAs were detected in insects [25] and it has been argued that plant miRNAs in animals are artefactual due to sequencing methodology. Some computational analyses also showed that plant-derived xenomiRs were divergent from reality [21, 25, 26]. For instance, Tosar [21] reported the amount of plant miRNAs in 3 human spermatozoa samples reached extreme, biologically meaningless level. Though it is still a controversial issue, the new findings about xenomiRs never stop. Emerging evidence suggests that the species of plant miRNAs detected in animals are limited, although the total species of miRNAs of a single plant is often more than several hundred, for example 713 species of miRNAs have been identified in *Oryza sativa* (osa) so far [27]. Only 25 species of plant miRNAs were detected by Zhang et al. [7], although their samples pooled 80 human serum (8 samples, each sample pooled from 10 humans). In another study of Zhang et al. [28], where plant miRNAs in human plasma were examined by qRT-PCR after donors had drunk fruit juice, 10 species of plant miRNAs were detected, whereas 16 species of plant miRNAs could be detected in the fruit juice. Similarly, limited species of maize miRNAs were detected using qRT-PCR in the serum and tissues of pigs feed with fresh maize for 7 days [9]. With TA-cloning and Sanger sequencing, only a part of species of mulberry-derived miRNAs were detected in hemolymphs of silkworms which were fed with mulberry leaves [11].

In fact, besides natural plant miRNAs, many species of synthetic plant miRNAs or mimic plant miRNAs that are identical or similar to natural plant miRNAs, were also reported to be able to transfer into and keep stable in animal bodies. Chin et al. [13] suggested that both natural plant miR159 and synthetic oligos, with the same sequence as miR159, were capable of transferring into human breast cancer cells. Similarly, the synthetic miR166b were detected in silkworm hemolymph [11]. A recent study reported that 3 species of mimic plant miRNAs (mmu-miR34a, mmu-miR143 and mmu-miR145) can also transfer into mouse body by oral administration [14]. Many reports suggested that MIR2911 could be significantly taken in by human and animals, which was attributed to its unique sequence [16, 29, 30], and the disruption of the MIR2911 sequence by two nucleotides abolished its absorption [31]. Yang et al. [30] suggested that not all miRNAs, but miRNAs with certain features could keep stable in GI tract of animals, and randomly synthesized miRNA-like sequences would be degraded quickly after injected in animals.

The discoveries described above imply the selective absorption of plant miRNAs by animals, i.e. only plant miRNAs with specific sequence could be absorbed by specific species of animals, which also provides an explanation for studies that reported the un-detectability [25, 32] of several plant miRNAs in animals. In this paper, we first systematically studied the sequence differences between the plant miRNAs which can transfer (xenomiR group) and cannot transfer (non-xenomiR group) into human bodies using statistics methods. Significant difference was found in 28 sequence features between the two groups, which suggested the potential patterns underlying the plant-derived xenomiR sequences and a possible link between these patterns with selective absorption of xenomiRs. Subsequently, a random forest (RF) model and a one-dimensional convolutional neural network (1D-CNN) model were trained to distinguish the two groups. Both models successfully distinguished between xenomiRs and non-xenomiRs with high accuracy. They were then used to predict potential plant-derived xenomiRs on unlabeled plant miRNAs, and a total of 241 plant miRNAs were identified as xenomiRs by both models. Finally, we analyzed the functions of the 241 predicted along with 166 previously reported xenomiRs in human body. Taken together, we report the first systematic plant-derived xenomiR sequences analysis, and the results provide evidence for selective absorption of plant miRNAs by human body. In addition, we propose the first list of high-probability xenomiRs, which further enables more robust decisions regarding plant miRNAs candidates for experimental validation and facilitates future investigation about the mechanisms of xenomiRs transferring into animal bodies.

## Results

### Datasets and feature extraction

For sequence comparison, we collected 166 xenomiR sequences (positive samples) and 942 non-xenomiR sequences (negative samples). All 166 xenomiRs (Additional file 1: Table S1) were collected from our previous study [33], which were obtained from 388 healthy human samples analyzed by a rigorous bioinformatics pipeline. These miRNAs covered almost all the reported plant-derived xenomiRs so far. Regarding non-xenomiRs (negative samples), no off the shelf dataset is available at present. To obtain the non-xenomiRs as accurately as possible, we carefully selected the miRNAs that have never been detected in human from *osa*, *Zea maize* (zma)*, Glycine max* (gma) and *Arabidopsis thaliana* (ath) (see “Methods”), which are either staple food or the plant closely related to common vegetables (see “Discussion”). In total, 942 miRNAs (Additional file 2: Table S2) were labeled as non-xenomiRs. For both positive and negative samples, we extracted the length, nucleotide positions, 1~ 3 nt motif frequency in full miRNA sequences and 1~ 2 nt motif frequency in miRNA seed regions (Additional file 3: Table S3). All these features are widely used in miRNA associated researches.

### Statistical analysis of the differences between xenomiRs and non-xenomiRs

**Fig. 1:**
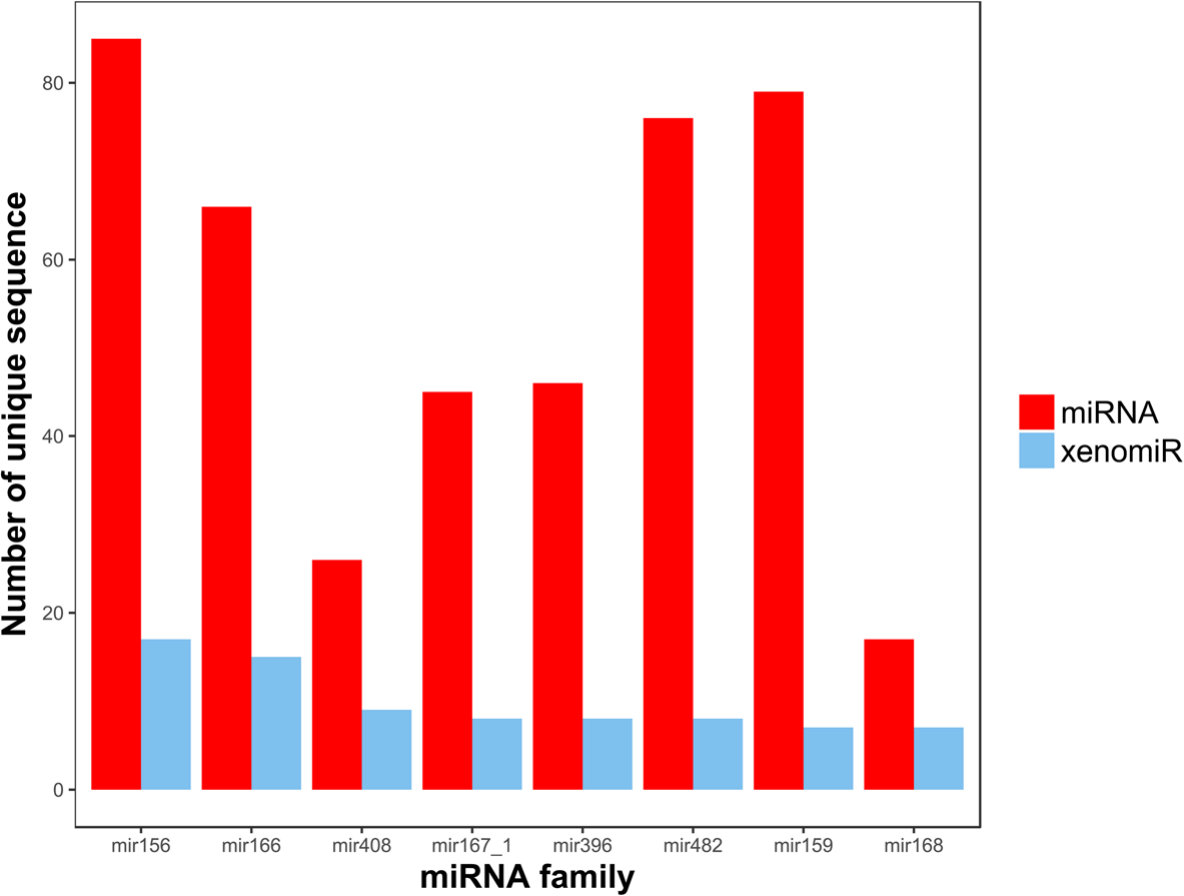
The top 8 plant miRNA families that contain the most xenomiRs. More than one half of xenomiRs belong to these 8 miRNA families. In mir168 family, up to 41.2% miRNAs (7 of 17 miRNA sequences) were mapped. All redundant sequences were removed from each RNA family

Considering the similarity of the members in the same miRNA family, we first studied the miRNA families to which the xenomiRs belong according to the miRNA families classified by miRBase [27]. In total, 49 miRNA families were mapped by xenomiRs (Additional file 4: Table S4), among which 8 (Fig. 1) covered more than half of the 166 xenomiRs. In mir168 family, up to 41.2% miRNAs (7 out of 17 miRNA sequences) were mapped by xenomiRs. These results suggested that xenomiRs are likely to enrich in specific miRNA families, rather than randomly distributed among all miRNA families. That may be caused by the common sequence features in miRNA families.

**Fig. 2:**
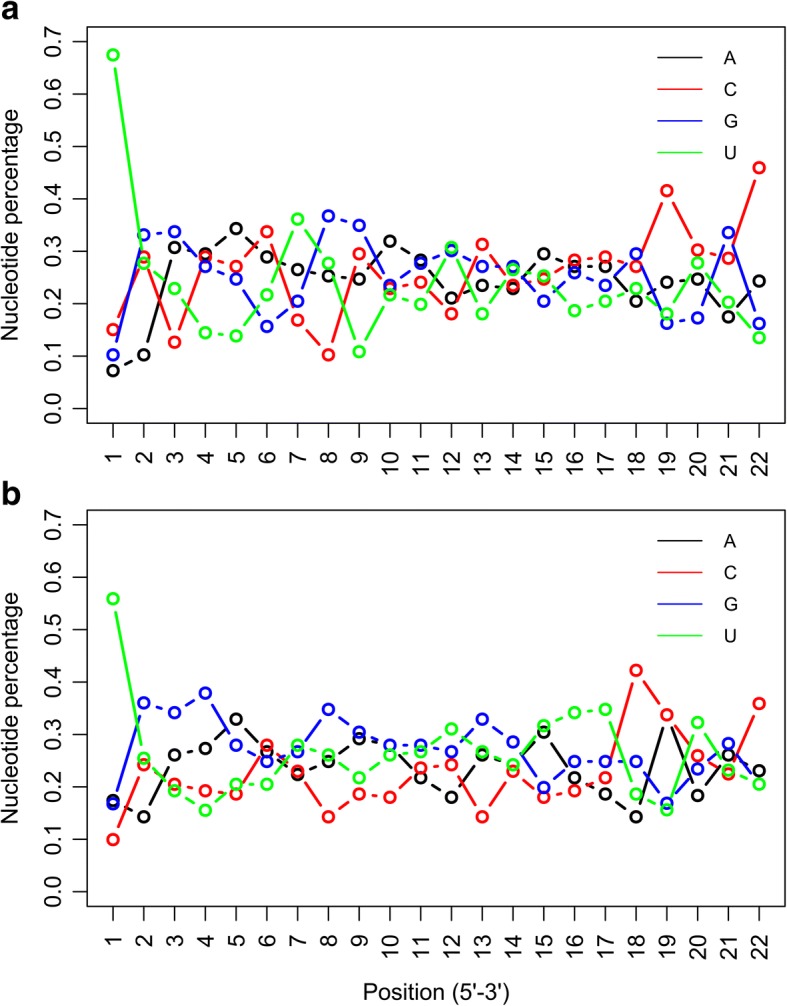
Nucleotide position comparison between xenomiRs and non-xenomiRs. Percentage of the four kinds of nucleotide at each position of **a**) 166 xenomiRs and **b**) 942 non-xenomiRs

Further, we explored the differences between xenomiRs (Additional file 1: Table S1) and non-xenomiRs (Additional file 2: Table S2) in terms of nucleotide position. The percentages of the 4 kinds of nucleotides (adenine (A), cytosine (C), guanine (G) and uracil (U)) in each position were obtained for each group (Fig. 2), respectively. It can be found that the percentages of nucleotides are different in xenomiRs and non-xenomiRs, especially the percentages of pyrimidines (U at the 1st, 7th, 9th, 13th, 15th~17th position, and C at the 3rd, 4th, 5th,6th, 9th, 13th, 15th~22nd position), suggesting the difference in position features between the two groups. Hypothesis tests were also performed on all the other features listed in Additional file 3: Table S3 for further comparison. In total, 23 out of 105 features were significantly different between the two groups (*p* < 0.01, false discovery rate (FDR) corrected), as listed in Table 1, including, C content, U content, six different 2-mer motifs, twelve kinds of 3-mer motifs and three kinds of 2-mer motifs in seed region. It can be found that, comparing with non-xenomiRs, the contents of most motifs (1~ 3 mer) with C nucleotides are higher in xenomiRs, yet the contents of most motifs with U nucleotides are lower. Besides, the sequence length of xenomiRs is also significantly shorter than that of non-xenomiRs. Taken together, our results suggested that xenomiRs and non-xenomiRs are separable in sequence feature space.

**Table 1:** Sequence feature comparison between xenomiRs and non-xenomiRs

Feature	*p* value (Wilcox-rank-sum test)	FDR	Mean (positive)	Mean (negative)
**C**	**1.27E-05**	**1.66E-04**	**0.254699729**	**0.206518506**
U	1.03E-03	5.16E-03	0.242165895	0.276586575
**AC**	**8.49E-04**	**4.69E-03**	**0.053561834**	**0.042817287**
AU	3.95E-04	3.13E-03	0.051751301	0.068725933
**CA**	**1.23E-10**	**6.46E-09**	**0.085152137**	**0.060351853**
**GC**	**1.00E-03**	**5.16E-03**	**0.065351632**	**0.056254644**
GU	2.35E-07	4.11E-06	0.034289003	0.057152059
UA	1.13E-09	3.97E-08	0.021754371	0.045343654
**AGC**	**2.00E-05**	**2.33E-04**	**0.026437639**	**0.016911021**
**CAC**	**1.27E-07**	**2.67E-06**	**0.019516663**	**0.010089057**
**CAG**	**5.85E-11**	**6.15E-09**	**0.027642769**	**0.013855275**
**CCC**	**2.41E-04**	**2.30E-03**	**0.016600043**	**0.007669506**
**GAA**	**1.32E-03**	**6.04E-03**	**0.028207567**	**0.022570777**
**GCA**	**8.94E-06**	**1.34E-04**	**0.027717889**	**0.016944631**
GGU	5.00E-04	3.27E-03	0.00755333	0.015033431
GUA	5.30E-04	3.27E-03	0.003583374	0.01049709
UAA	4.42E-04	3.13E-03	0.003479138	0.010353066
UAU	7.81E-09	2.05E-07	0.001510348	0.013291427
UUA	4.47E-04	3.13E-03	0.003682193	0.01009223
UUU	1.09E-03	5.18E-03	0.010166663	0.021144843
GU (seed)	4.75E-05	4.98E-04	0.031124498	0.060686483
**GA (seed)**	**3.27E-04**	**2.86E-03**	**0.123493976**	**0.088110403**
UA (seed)	8.28E-04	4.69E-03	0.016064257	0.037508846

Feature comparison was performed between xenomiRs and non-xenomiRs, and 23 features with FDR less than 0.01 were listed. Bold indicates the values are higher and non-bold indicates lower in xenomiRs than non-xenomiRs

**Fig. 3:**
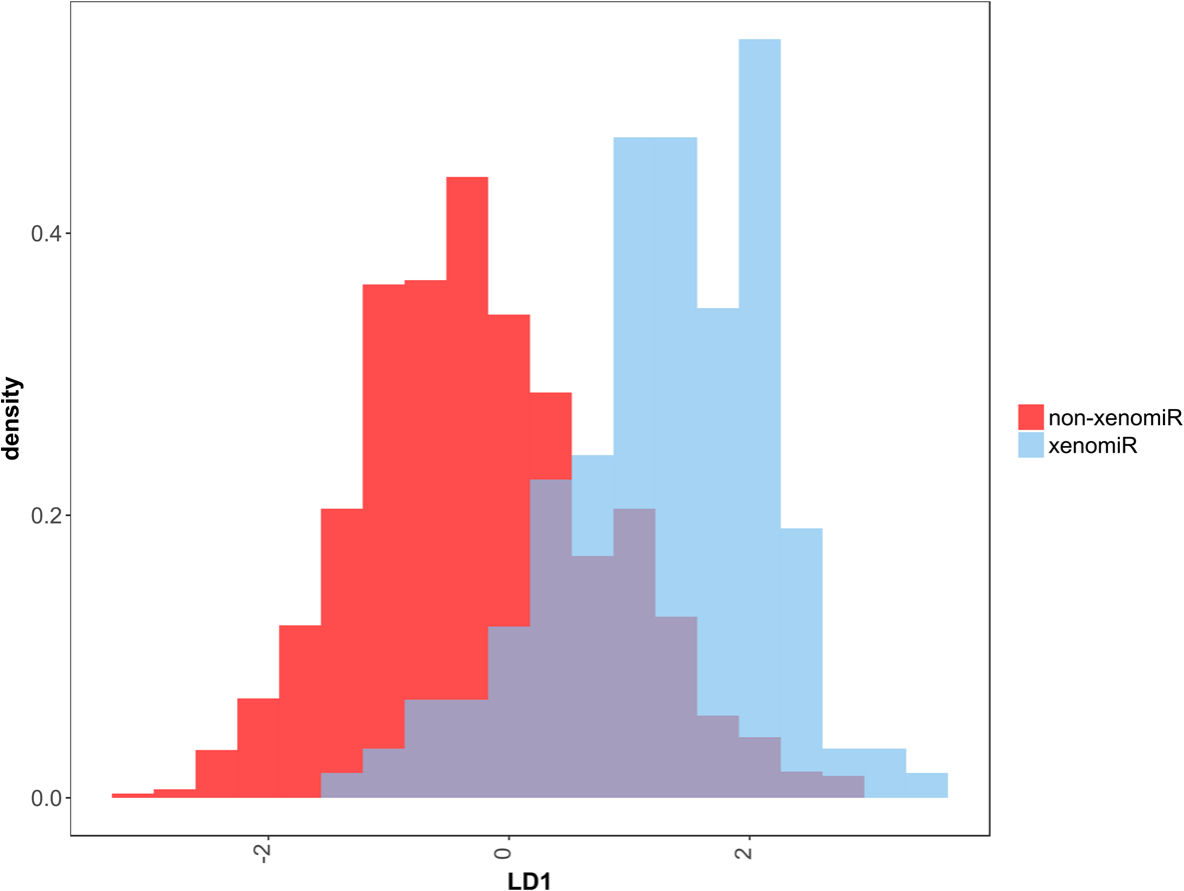
Dimension reduction of features extracted from xenomiRs and non-xenomiRs. Dimension reduction of features extracted from xenomiRs and non-xenomiRs was performed using LDA to show the differences between them. Overall, the xenomiRs and non-xenomiRs could be separated with partial overlap in the middle, and the distribution of xenomiRs is more compact than that of non-xenomiRs

To easily observe the multi-dimensional feature differences between xenomiRs and non-xenomiRs, linear discriminant analysis (LDA) was performed to visualize the differences in lower dimension. We selected all features except position features listed in Additional file 3: Table S3 to describe miRNA sequences, and the density of LD1 was shown in Fig. 3. Overall, the xenomiRs and non-xenomiRs could be separated with partial overlap in the middle, and the distribution of xenomiRs is more compact (*p* < 2.2e-16, see “Methods”) than that of non-xenomiRs. To better distinguish xenomiRs and non-xenomiRs for accurately predicting potential xenomiRs, two more complicated models, random forest and one-dimensional convolutional (1D-CNN) neural network were employed.

### Model building and training

**Fig. 4:**
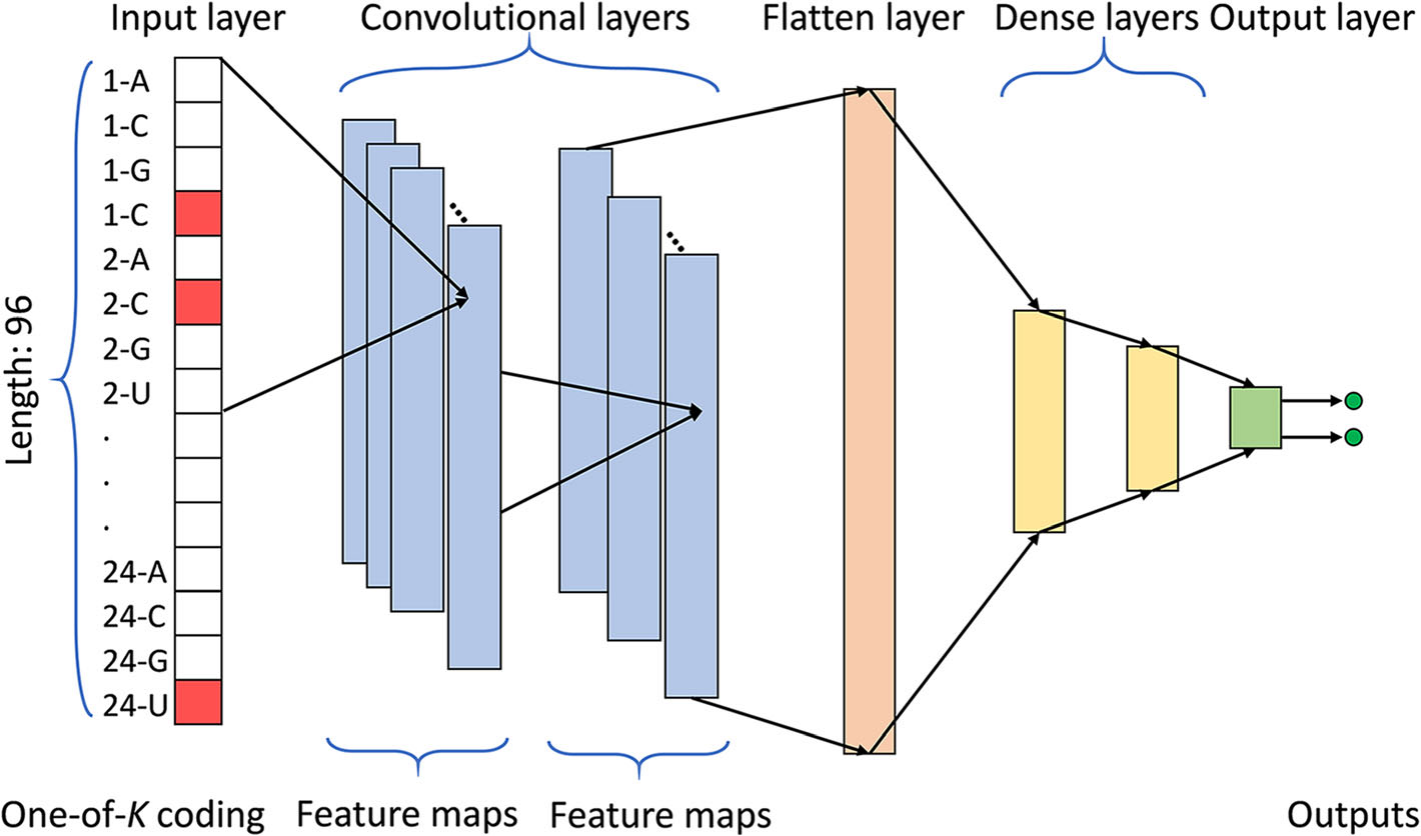
The architecture of our 1D-CNN model. This model consists of two convolutional layers, one flatten layer, two dense layers and one output layer

In our RF model, 1~ 3 mer motifs in full miRNA sequence, 1~ 2 mer motifs in seed region and the length of miRNA were employed as inputs. The number of decision trees and the number of features randomly sampled as candidates at each split were set to 501 and 6, respectively. The framework of our 1D-CNN is summarized in Fig. 4 (see “Methods”), which contained 2 convolutional layers, 1 flatten layer, 2 dense layers and 1 output layer. We encoded the four kinds of nucleotides by one-of-*K* fashion, and for each miRNA sequence, the codes of the first 18 nucleotides of a raw miRNA sequence were flattened into a one-dimensional vector, which was used as inputs (see “Methods”). Furthermore, L2 regulation and dropout [34] strategy were employed to relieve 1D-CNN model from overfitting, and the hyper-parameters used in our 1D-CNN model were determined using Bayesian optimization method.

### Performance evaluation

An independent test set containing 25 positive samples (15% of all positive samples) and 25 negative samples, which were randomly selected from positive and negative samples, was used only in testing process, and all the other samples consisted of the training set (Table 2). To deal with the imbalance between positive and negative samples in training set (141 versus 917), an oversampling strategy was employed (see “Methods”).

**Table 2:** Training set and testing set

Data set	Class	# of miRNAs	Data source
Training	Positive	141	Literature [33]
	Negative	917	miRbase, GEO
Testing	Positive	25	Literature [33]
	Negative	25	miRbase, GEO

An independent test set containing 25 positive samples (15% of all positive samples) and 25 negative samples, which were random selected from positive and negative samples, was used only in the testing process, and all the other samples were used for training.

**Fig. 5:**
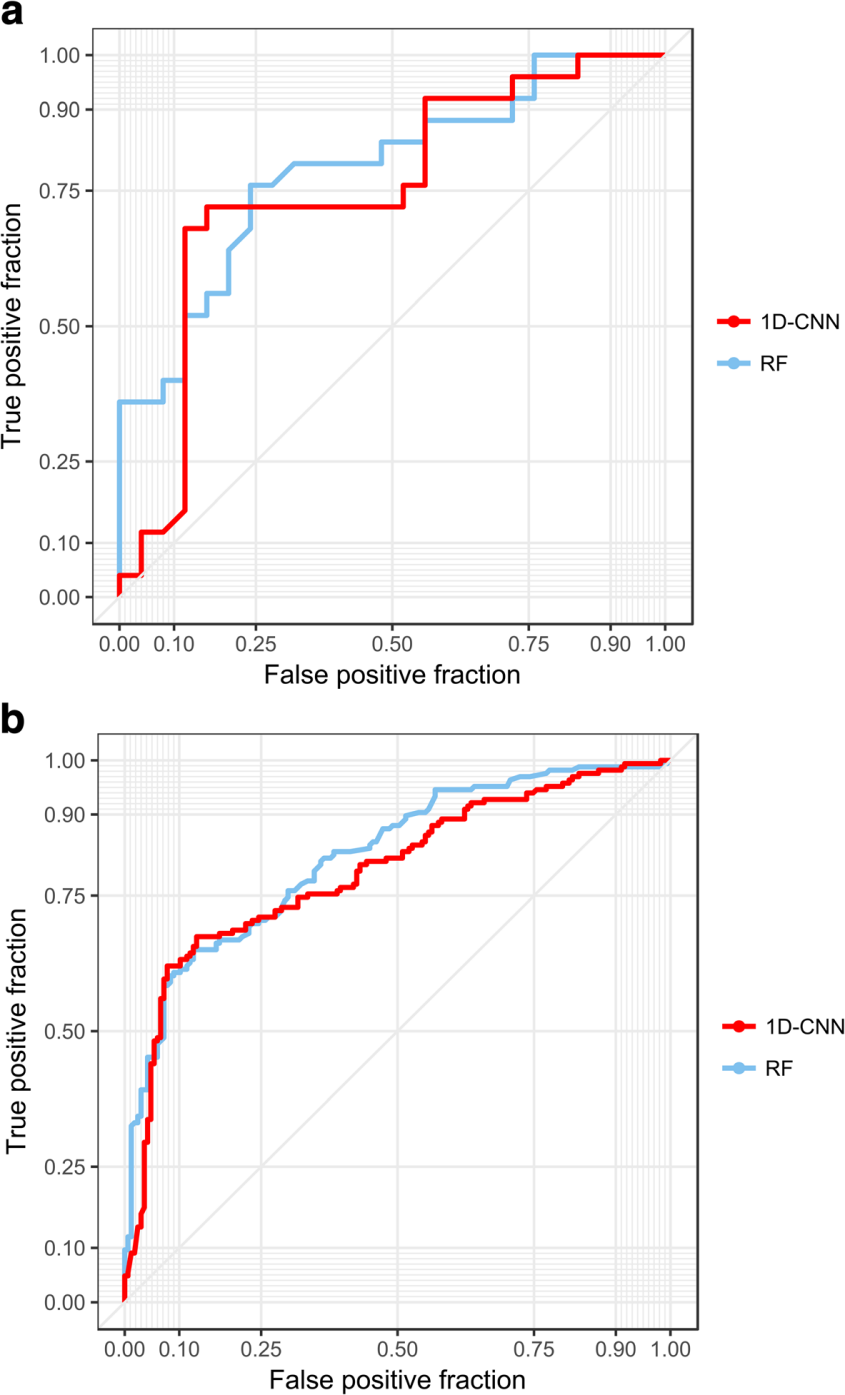
ROC curves for performance comparison. ROC curves for performance comparison between RF and 1D-CNN models by (**a**) test set and (**b**) 5-fold cross validation, respectively

To compare model performance, our RF model and 1D-CNN model were trained and tested under the same training set and testing set. As shown in Table 3, both models achieved relatively high accuracy, and their performance was comparable. The RF model achieved better SN (0.920), at the cost of lower SP (0.560). The SN of 1D-CNN model (0.880) is lower than that of random forest model, however, the specificity is much higher (0.680). In the meantime, our 1D-CNN model achieved higher ACC (0.780) and MCC (0.574) than RF model, but the AUC under the ROC curve is lower (0.817) (Fig. 5a). To further ensure that our models were independent of training and testing sets, a 5-fold cross-validation was performed. Comparable results were obtained for both RF and 1D-CNN models, as shown in Fig. 5b and Table 3.

**Table 3:** Accuracy comparison between RF model and 1D-CNN model on test set and 5-fold cross-validation set

Model		SN	SP	ACC	AUC	MCC
Independent test set	RF	0.920	0.560	0.740	0.866	0.547
	1D-CNN	0.880	0.680	0.780	0.817	0.574
5-fold CV	RF	0.901	0.666	0.736	0.840	0.538
	1D-CNN	0.859	0.712	0.766	0.794	0.543

*ACC, SN, SP, AUC* and *MCC* indicate accuracy, sensitivity, specificity, area under the *ROC*

curve and Mathews correlation coefficient, respectively

We further obtained the top 10% most important features evaluated by mean decrease accuracy and mean decrease Gini obtained from our RF model (Additional file 5: Table S5). The important features identified by both methods are highly consistent (7 out of 10) and also in line with the features that show significant difference between the xenomiRs and non-xenomiRs (Table 1). Among them, the motif ‘CAG’ was evaluated as the most important feature by mean decrease Gini, and it was also the only 3 mer motif feature evaluated by mean decrease accuracy method.

### Prediction of potential xenomiRs from unlabeled plant miRNAs and xenomiR potential target analysis

**Fig. 6:**
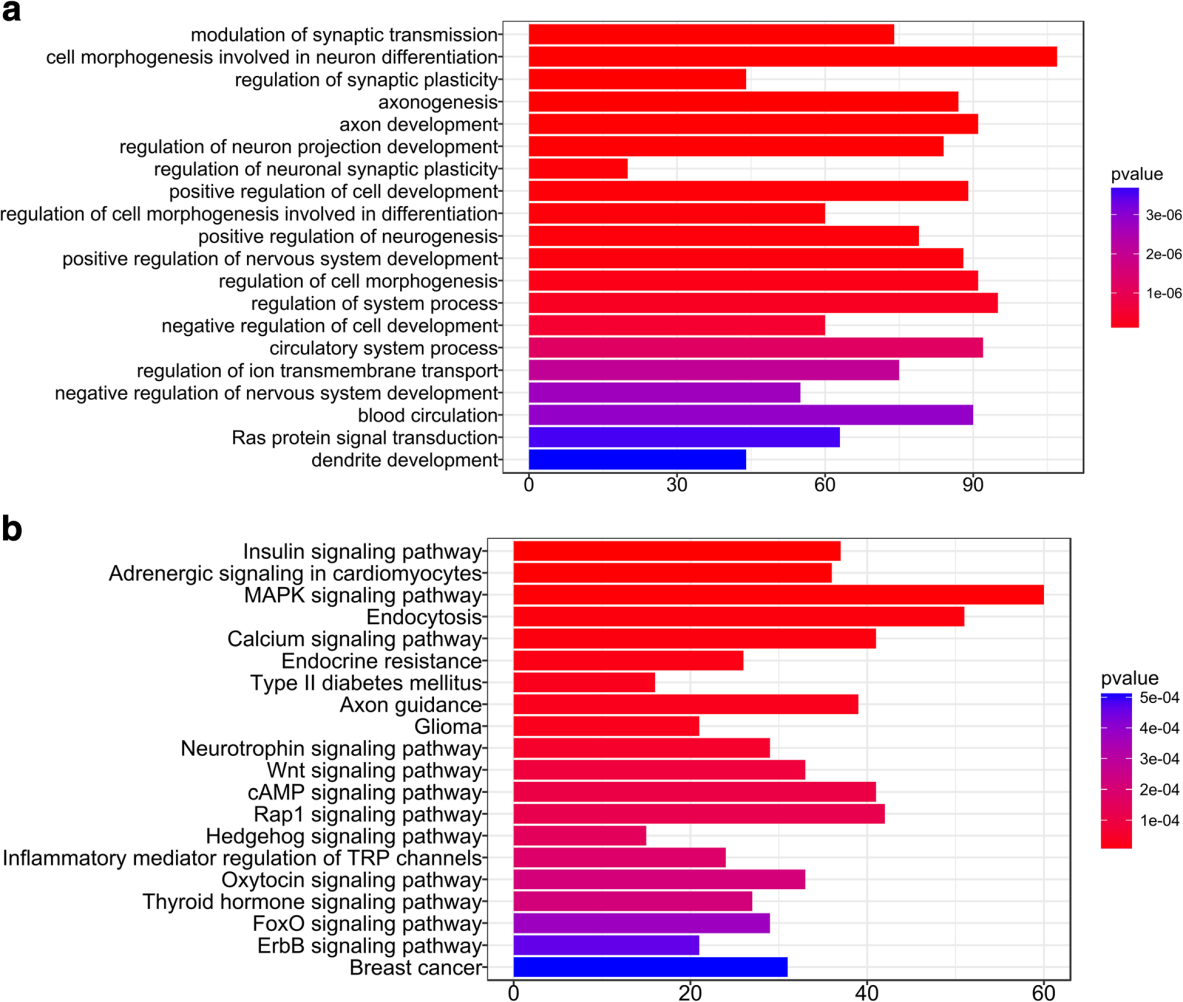
Enriched biological processes and KEGG pathways. The top 20 enriched biological processes (**a**) and the top 20 KEGG pathways (**b**) shown by Gene Ontology analysis pathway enrichment analysis, respectively

Both RF model and 1D-CNN models were used for predicting potential xenomiRs from all unlabeled 3695 plant miRNAs (Additional file 6: Table S6) with unique sequences in miRBase [27] (see “Methods”). In total, 643 and 555 miRNAs were predicted as xenomiRs by RF and 1D-CNN models, respectively, and 241 miRNAs (Additional file 7: Table S7) were predicted by both models (Additional file 8: Figure S1). Being conservative, we only considered these 241 miRNAs as predicted xenomiRs. Further, we analyzed the potential functions of all possible xenomiRs in human bodies, including 166 previously reported xenomiRs (Additional file 1: Table S1) and 241 predicted xenomiRs (Additional file 7: Table S7). Specifically, we firstly obtained the potential target genes of xenomiRs using miRanda [35] and RNAhybrid [36], which are commonly used miRNA target prediction tools. The 2194 unique target genes (Additional file 9: Table S8) identified by both tools were regarded as high-probability targets (see “Methods”). Subsequently, gene ontology analysis was employed to annotate the biological processes enriched by the target genes (see “Methods”). The top 20 most significantly enriched biological processes were shown in Fig. 6a. It can be seen that the target genes are likely related to the development, differentiation, regulation of neural system, and the regulation of circulation system. Similarly, pathway enrichment analysis was employed to find potential biological pathways involved by xenomiRs, and the top 20 most significantly enriched pathways were shown in Fig. 6b. Results indicated that the target genes are related to endocrine, cancer and inflammatory regulation pathways.

## Discussion

We have conducted the first systematic analysis of sequence differences between xenomiRs and non-xenomiRs, and significant difference was found, which argued in favor of the selective nature of the absorption of xenomiRs and its relation with miRNA sequences. We have then shown the feasibility of distinguishing between xenomiRs and non-xenomiRs based on miRNA sequences using machine learning models. High accuracies were achieved by both random forest model and 1D-CNN model. This could serve as a valuable tool for predicting potential xenomiRs that have not yet been discovered, based on which further biological experiments could be conducted for validating their ability of transferring into human bodies and more importantly, exploring their potential functions. The important features of xenomiRs identified here might offer insights into underlying mechanisms of xenomiRs transferring into and keeping stable in animal body. In addition, we have provided the first list of high-probability xenomiRs as well as their potential functions. Of interest, the functions of the predicted targets of these xenomiRs seem to be consistent with previous studies (see details below). We have shown in our previous paper [33] that the plant miRNAs detected in human bodies are tissue-specific and cannot be fully explained by contamination and provided evidence for the xenomiRs hypothesis. The selective absorption of plant miRNAs by animal bodies could provide an explanation for studies where xenomiRs were not detected in animal bodies. If more plant-derived xenomiRs in different human tissues are available, our 1D-CNN model could be adjusted slightly using transfer learning [37] to learn the different patterns of plant-derived xenomiRs in different tissues. Thus, the channel tropism proposed in Inner Gannon of Huangdi [38] may be better explained, and the corresponding tissues, where a specific herb function may also be predicted.

More than one hundred species of xenomiRs have been reported so far, however, more species of xenomiRs remain to be discovered, especially those in the plants seldom consumed, such as traditional medical herbs. The discovery of xenomiRs in traditional medical herbs has great significance in better understanding of the mechanisms underlying the therapeutic function of these herbs. Therefore, to predict the potential xenomiRs using machine learning model is of great significance. However, since no off the shelf data were available, we collected xenomiRs/non-xenomiRs in the following way.

The positive dataset was extracted through a rigorous pipeline, which includes all plant-derived miRNAs identified from all sRNA-seq data of healthy individuals in NCBI GEO database (before 2016). It accorded well with the xenomiRs reported in human tissues [33]. Hence, the positive dataset contained currently reported plant-derived xenomiRs in common food. It is likely that some plant-derived xenomiRs were not included in the positive dataset due to their low abundance under normal condition. However, it is still the optimum choice given current scientific advances.

The collection of negative data, on the other hand, is less straightforward. We chose miRNAs from plants that are human everyday diet or closely related to common vegetables, but have never been detected in human samples. There might exist xenomiRs remain undetected, due to either the inability of current detection technique or inappropriate experimental conditions, such as the time after oral administration or amount of food taken in. To maximally avoid false negatives in our negative dataset, we included only “non-xenomiRs” in four species that can be regarded as everyday diet, i.e. ath, gma, osa and zma (ath is closely related to common vegetables, such as *Brassica rapa*, *Brassica oleracea*, *Brassica juncea* and *Oilseed rape*). Therefore, the selection of negative dataset is based on current advances in plant-derived xenomiRs. Of note, we cannot rule out all false negatives, which might limit the accuracy of our models.

However, the identification of these false positives/negatives requires biological verification, which is time consuming due to the enormous number of candidates. Our model, although imperfect, could serve as the first and efficient tool for selecting the most probable ones for further experimental verification. The verified xenomiRs or non-xenomiRs could, in turn, be fed to our model, and thus improve its accuracy.

The 166 xenomiRs in the positive dataset are from 49 miRNA families and 55.3% are from 8 miRNA families (Additional file 10: Table S10). For non-xenomiRs in the negative dataset, 48% are from these 49 miNRA families but only 19.0% are from the top 8 families containing xenomiRs (Additional file 10: Table S10). In the prediction set, 201 (83.4%) additional members of these 49 miRNA families have been predicted as xenomiRs, among which 123 (50.9%) are from the top 8 families (Additional file 10: Table S10). It can be seen that both collected and predicted xenomiRs concentrate in limited and similar group of families.

In feature extraction, each sequence was assumed to have 24 nt. The choice of 24 nt was not arbitrary. We tried to use the fist 21 nt ~ 23 nt as well as the last 21 nt ~ 23 nt of each miRNA sequence as the sequence position features, for better capturing 5′ end and 3′ end absolute sequence position features, respectively. The final choice was made upon the model performance, i.e. training our models using 24 nt could slightly outperform the other cases. Furthermore, only 0.047% plant miRNAs for prediction are longer than 24 nt. Thus, truncating the part of miRNAs longer than 24 nt had minor effect on the performance of our models. We chose sequence-based features because they are very commonly used to characterize miRNAs and play essential roles in the function of miRNAs. They might also affect miRNAs’ ability of entering human body. This motivated us to investigate the difference in sequence-based features between xenomiRs and non-xenomiRs. Our results, indeed, confirmed that existence of significant difference between these two groups of miRNAs (Table 1).

The structure-based features of miRNAs were also tried to use in training our models. However, because a miRNA is very short, most of them cannot construct a stable secondary structure, which was verified by predicting miRNA secondary structures using both RNAfold [39] and our previously published tool Fledfold [40, 41]. Nevertheless, adding secondary features of pre-miRNA, including stem features, hairpin loop features, bulge loop features, internal loop features and energy (stability), did not improve model performance. There are two possibilities resulting in this. First, the ability of miRNA entering human body has little to do with the secondary structure of a pre-miRNA. Alternatively, the sample size might limit the usefulness of additional features given to the model.

In our study, the positive dataset was composed of miRNAs from more than 20 species, among which 57% were from ath, gma, osa or zma. All the negative miRNAs were from these four species. Therefore, it is very unlikely that the models simply perform species recognition. If the dataset were separated by species (ath, gma, osa and zma), the positive dataset will be very small (average 24 miRNAs for each specie), leading to a high risk of overfitting. A model such like that has poor generalization ability. Re-training our models using miRNA data from single species (ath, gma, osa or zma) verified this thought: the re-rained model performs much better on training set, compared to testing set (accuracy: 81% VS 62%).

High GC content was reported to be responsible for the absorption of MIR2911 by animal bodies [29]. More generally, the high GC content [16] and the short length could increase the stability of an RNA. Our results confirmed this by systematic statistical analysis, revealed that xenomiRs have higher GC content (*p* < 0.05) and shorter length (p < 0.05), compared to non-xenomiRs. Meanwhile, other differences between xenomiRs and non-xenomiRs sequences were also identified (Fig. 2, Table 1). Besides, the important features obtained from our RF model (Additional file 5: Table S5) provided more insights into the patterns of xenomiR sequence. And if some important patterns are sabotaged, the absorption might be abolished, as in the case where miR2911 sequence is disrupted by just two GC nucleotides [31]. In addition, the 3 mer motif ‘CAG’ was evaluated as one of the most important patterns for distinguishing xenomiRs with non-xenomiRs (Additional file 5: Table S5), suggesting its possible relation with the transference or stability of xenomiRs.

These results supported our assumption that plant-derived miRNAs are absorbed selectively by human and other animals, and only the sequence of a miRNA with certain patterns could be transferred into human bodies. However, further studies are needed to identify more concrete patterns in xenomiR sequences.

Deep Learning has been widely applied in bioinformatics and obtained satisfactory performance [42, 43]. Our study used a 1D-CNN model to identify xenomiRs using only raw miRNA sequences as inputs, and higher prediction accuracy was achieved on independent test set, compared to RF model, which used 105 hand-craft features as inputs. It is likely that 1D-CNN model, which is capable of extracting the features of successive nucleotides, could capture the specific patterns underlying xenomiR sequences that contain important information for identifying xenomiRs.

To uncover the potential role of xenomiRs in human body, we analyzed the function of predicted targets of xenomiRs using enrichment analysis tools [44]. Many identified functions have already been reported by other studies with bio-experiments. For example, xenomiRs were reported to be related with cancer [13, 14], inflammatory [16, 18], circulation system [7] in human body. And recent studies reported an association between xenomiRs with neuron development of pandas [10], and the caste development of honeybees [45].

This study does not deny the animal-derived xenomiRs hypothesis. However, animal-derived xenomiRs are much more difficult to identify because of the high sequence conservation, which obscures the differences between dietary animal miRNAs and endogenous miRNAs [8]. Hence, in this study, we only studied the plant-derived xenomiRs.

In addition, many other factors could affect the detectability of xenomiRs in human samples, such as, the miRNA abundance in the consumed plant materials, the stability of plant miRNAs, the time after consumption [28] and some special molecules in the diet consumed along with plant miRNAs [18]. Therefore, in xenomiR studies, randomly selecting miRNAs to perform biological verification is risky. The methodology present in this paper could serve as a valuable and efficient tool for selecting candidate plant miRNAs for biological verification.

## Methods

### Data sets

We collected 166 xenomiRs reported in our previous study as positive data set [33]. To obtain reliable negative data set, we collected small RNA sequencing samples of *ath*, *gma*, *osa*, *zma* using GEO database [46] and miRBase [27]. Specifically, to decrease the false negatives in the negative dataset, the miRNAs were carefully selected. Some miRNAs are only expressed at extreme conditions, such as drought. Hence, instead of directly selecting the non-xenomiRs in four species of plants (ath, gma, osa, zma) from miRBase, we collected non-xenomiRs from 12 sRNA sequencing data in NCBI GEO database. For convenience, we used the processed data provided by GEO. Firstly, the miRNA reads were screened out using miRBase, and abundance of reads in the last 30% were removed. They are probable false negatives because their undetectability in human body might be due to the rather low abundance, and removing those miRNAs with low abundance could reduce possible false negatives in the negative dataset. Furthermore, we removed the miRNAs contained in the positive dataset. The remaining miRNAs constituted non-xenomiRs set for each species were obtained. We merged the miRNA sets obtained from different collected samples, resulting in a pooled miRNA set of four species of plants. In total, 942 non-xenomiRs were obtained (Additional file 1: Table S1) from the pooled miRNA set. Each miRNA sequence was unique in either positive or negative dataset. Besides, removing the miRNAs labeled as xenomiRs and non-xenomiRs in this study, the remaining miRNAs (3695) in miRBase [27] with unique sequences and length more than 18 (containing 18) nt were regarded as unlabeled samples and used for xenomiR prediction.

### Feature extraction

In total, 129 features were extracted from miRNA sequences, which were listed in Additional file 3: Table S3, including sequence length, nucleotide position, 1~ 3 met motif frequency in both full miRNA sequences and miRNA seed region (2nd ~ 8th nt). In 1D-CNN model, four kinds of nucleotide (A, C, G, U) were represented by one-of-*K* (*K* = 4) coding, i.e., binary code ‘0001’ for A, ‘0010’ for C, ‘0100’ for G and ‘1000’ U. Each sequence was assumed to have 24 nt. The RNAs with the length less than 24 nt were filled with code ‘0000’ at the end of miRNAs, whereas the RNAs with longer sequence were truncated. This is because in both collected positive and negative dataset, all miRNAs have a length no longer than 24 nt. Besides, a miRNA is labeled with 1 if it is a xenomiR or 0 otherwise.

#### LDA

LDA was performed using 1~ 3 mer motifs in full miRNA sequence, 1~ 2 mer motifs in seed region and the length of miRNA. LD1 for each sample was obtained, and its distribution for both xenomiR and non-xenomiR groups were shown in Fig. 3. To compare the degree of compactness for LD1 distribution of the two groups, any distance of LD1 (LD1 distance) between two sample within both groups was obtained, and t-test was performed to test the difference between LD1 distances of the two groups.

### Performance measures

Commonly used metrics were used to evaluate the performance of our models, namely accuracy (ACC), sensitivity (SN), specificity (SP) and Matthews correlation coefficient (MCC), of which the formulas were summarized in Additional file 11: Table S9. Receiver operating characteristic (ROC) curves were plotted using SN and SP, and areas under ROC curves (AUC) were also calculated to further compare the performance of our models.

### One dimensional CNN

Keras framework (https://keras.rstudio.com) was employed to build our 1D-CNN model. The one-of-*K* coding of first *L* nucleotides were flattened into a single one-dimensional vector as inputs for our 1D-CNN model. Since the length of most plant miRNA sequences in xenomiRs or non-xenomiRs are more than 18 nt, *L* was set to 18. Hence, *L* × *K* units were used in the input layer. Our 1D-CNN model consisted of two convolutional layers to extract features from the miRNA sequences. After flatting the feature maps of second convolutional layer, two dense layers were used, which employed dropout technique and L2 regulation to avoid overfitting. All the layers used sigmoid function as activation function except the two-unit output layer, where softmax function was used. Bayesian optimization was employed to optimized channel sizes and kernel sizes in each convolutional layer, number of units in dense layers, dropout rate and lambda in L2 regulation.

To deal with the imbalance between positive and negative samples in the dataset, an oversampling strategy was employed. Given the training samples containing *P* positive samples and *N* negative samples (*P* < < *N*), oversampling strategy is as follows. The positive samples were extended to the number of *N* by random sampling in *P* samples with replacement, meanwhile, all the positive samples should be sampled at least one time, resulting in the same number of positive and negative samples. Hence, the same number (*N*) of positive and negative samples were obtained in the training process. When performing the cross validation, the oversampling process was conducted inside the validation loop, i.e., oversample the minority class after the validation set has already been removed from the training set. Thus, the samples in the validation set will not be duplicated and further used in the training process. Doing this way, overfitting of the model is avoided.

### Targets prediction of plant-derived xenomiRs and enrichment analysis

We assumed that plant-derived xenomiRs can suppress the target genes in a working manner of endogenous miRNAs. Human 3’ Untranslated Regions (3’ UTR) sequences were downloaded from UCSC Genome Browser database [47]. Miranda [35] and RNAhybrid [36] were employed to predict the target genes of xenomiRs, both of which are widely used in miRNA target prediction. And the unique target genes predicted by both tools were regarded as potential plant-derived xenomiR targets (S8 Table). Corresponding gene names were collected for further annotation analysis and GO annotation. KEGG pathway were performed for identifying significant enriched (FDR < 0.01) biological processes and pathways using “clusterProfiler” package [44].

## Conclusion

Taken together, this study showed the sequence differences between xenomiRs and non-xenomiRs, and provided the first insights into the sequence specificity of xenomiRs. This could facilitate our better understanding of mechanisms underlying the absorption of plant-derived xenomiRs, as well as the biological processes participated. In addition, we showed the feasibility of using machine learning models for predicting potential plant-derived xenomiRs based on miRNA sequences and made the first attempt to build such models. Furthermore, this study showed that, in xenomiR studies, randomly picking plant miRNAs to carry out a bio-experiment could be risky, in terms of being inefficient, and the plant miRNAs should be decided with great care, for example, picking miRNAs in detected xenomiRs (Additional file 1: Table S1) or predicted xenomiRs (Additional file 7: Table S7) provided in our study.

## Additional files

Additional file 1: **Table S1.** Positive samples. (DOCX 20 kb)

Additional file 2: **Table S2.** Negative samples. (DOCX 50 kb)

Additional file 3: **Table S3.** Feature list. (DOCX 14 kb)

Additional file 4: **Table S4.** The RNA families mapped by xenomiRs. (XLSX 10 kb)

Additional file 5: **Table S5.** The top 10% most important features evaluated by RF model using mean decrease accuracy (left) and mean decrease Gini (right), respectively. (DOCX 14 kb)

Additional file 6: **Table S6.** All unlabeled 3695 plant miRNAs in miRBase. (XLSX 108 kb)

Additional file 7: **Table S7.** The 241 potential miRNAs predicted by both RF model and 1D-CNN model. (DOCX 23 kb)

Additional file 8: **Figure S1.** Venn diagram showed the 241 potential xenomiRs predicted by both RF and 1D-CNN models. (XLSX 43 kb)

Additional file 9: **Table S8.** The 2194 unique target genes predicted by both miRanda and RNAhybrid. The genes were encoded in entrez ID. (TIF 843 kb)

Additional file 10: **Table S10.** miRNA family table. (XLSX 18213 kb)

Additional file 11: **Table S9.** Accuracy measurement. (DOCX 22 kb)

## Abbreviations

1D-CNN: one-dimensional convolutional neural network
A: Adenine
ACC: Accuracy
ath: *Arabidopsis thaliana*
AUC: Areas under ROC curve
C: Cytosine
FDR: False discovery rate
G: Guanine
GI: Gastrointestinal
gma: *Glycine max*
LDA: Linear discriminant analysis
osa: *Oryza sativa*
RF: Random forest
RICS: RNA-induced silencing complex
ROC: Receiver operating characteristic
SN: Sensitivity
SP: Specificity
U: Uracil
xenomiR: Exogenous miRNA
zma: *Zea maize*
