# Supplementary material for: Prediction of plant-derived xenomiRs from plant miRNA sequences using random forest and one-dimensional convolutional neural network models
Source: BMC Genomics. 2018 Nov 26;19:839. doi: 10.1186/s12864-018-5227-3 (PMC6258294; doi:10.1186/s12864-018-5227-3)
Supplement: Supplementary file 1 — Table S1. Positive samples. (DOCX 20 kb) [file 12864_2018_5227_MOESM1_ESM.docx]

| **No.** | **Sequence** |
| --- | --- |
| 1 | UCGAUAAACCUCUGCAUCCAG |
| 2 | UUGGACUGAAGGGAGCUCCCU |
| 3 | UUCCACAGCUUUCUUGAACUG |
| 4 | UGACAGAAGAGAGAGAGCAC |
| 5 | UUGACAGAAGAUAGAGAGCAC |
| 6 | UUGACAGAAGAGAGAGAGCAC |
| 7 | UUUGGAUUGAAGGGAGCUCUA |
| 8 | UGAAGCUGCCAGCAUGAUCUU |
| 9 | UUGGCAUUCUGUCCACCUCC |
| 10 | AUGCACUGCCUCUUCCCUGGC |
| 11 | UGACAGAAGAGAGUGAGCAC |
| 12 | CGACAGAAGAGAGUGAGCAC |
| 13 | UGACAGAAGAUAGAGAGCAC |
| 14 | UCCCAAAUGUAGACAAAGCA |
| 15 | UCCCAAAUGUAGACAAAGC |
| 16 | UUUGGAUUGAAGGGAGCUCUU |
| 17 | UUUGGAUUGAAGGGAGCUCCU |
| 18 | UUGAAAGUGACUACAUCGGGG |
| 19 | UGGAGAAGCAGGGCACGUGCA |
| 20 | UGGAGAAGCAGGGCACGUGCG |
| 21 | UCGGACCAGGCUUCAUUCCCC |
| 22 | GGAAUGUUGUCUGGCUCGAGG |
| 23 | UGAAGCUGCCAGCAUGAUCUA |
| 24 | UGAAGCUGCCAGCAUGAUCUGG |
| 25 | CCCGCCUUGCAUCAACUGAAU |
| 26 | UCGCUUGGUGCAGGUCGGGAA |
| 27 | CAGCCAAGGAUGACUUGCCGA |
| 28 | CAGCCAAGGAUGACUUGCCGG |
| 29 | AGAAUCUUGAUGAUGCUGCAU |
| 30 | AGAAUCUUGAUGAUGCUGCAG |
| 31 | GAAUCUUGAUGAUGCUGCAU |
| 32 | UUCGCUUGCAGAGAGAAAUCAC |
| 33 | AAGCUCAGGAGGGAUAGCGCC |
| 34 | CUGAAGUGUUUGGGGGAACUC |
| 35 | GUUCAAUAAAGCUGUGGGAAG |
| 36 | GCUCAAGAAAGCUGUGGGAAA |
| 37 | UUCCACAGCUUUCUUGAACUU |
| 38 | UCAUUGAGUGCAGCGUUGAUG |
| 39 | UUAGAUUCACGCACAAACUCG |
| 40 | CAGGGAACAAGCAGAGCAUGG |
| 41 | CUGACAGAAGAGAGUGAGCAC |
| 42 | UCGGACCAGGCUUCAUUCCUC |
| 43 | UCGGACCAGGCUUCAUUCCU |
| 44 | UGAUUGAGCCGUGCCAAUAUC |
| 45 | UGUGUUCUCAGGUCGCCCCUG |
| 46 | UCUUGCCGACUCCUCCCAUACC |
| 47 | UCAGGAGAGAUGACACCGACG |
| 48 | GCUCAUUUCUCUCUCUGUCAGC |
| 49 | UGGAGAAGCAGGGCACGUGCU |
| 50 | GGAAUGUUGUCUGGUUCAAGG |
| 51 | GGAACGUUGGCUGGCUCGAGG |
| 52 | AGGUCAUGCUGGAGUUUCAUC |
| 53 | UGAAGCUGCCAGCAUGAUCUGA |
| 54 | CCCGCCUUGCACCAAGUGAAU |
| 55 | UCGCUUGGUGCAGAUCGGGAC |
| 56 | UGAAGUGUUUGGGGGAACUC |
| 57 | UCCACAGGCUUUCUUGAACUG |
| 58 | GUUCAAUAAAGCUGUGGGAAA |
| 59 | UGGAAGGGGCAUGCAGAGGAG |
| 60 | UGAGAAGGUAGAUCAUAAUAGC |
| 61 | CCCCAAAUGUAGACAAAGCA |
| 62 | UCGGACCAGGCUUCAUCCCCC |
| 63 | CGAAACUGGUGUCGACCGACA |
| 64 | ACAGGGAACAAGCAGAGCAUG |
| 65 | GAUCCCCGGCAACGGCGCCA |
| 66 | UUAGAUGACCAUCAACAAACU |
| 67 | UCUCGGACCAGGCUUCAUUCC |
| 68 | UGAAGCUGCCAGCAUGAUCUG |
| 69 | UUGGACUGAAGGGAGCUCCC |
| 70 | UGACAACGAGAGAGAGCACGC |
| 71 | UUCAGGAGAGAUGACACCGACA |
| 72 | GCUCACUUCUCUCUCUGUCAGC |
| 73 | UUUGGAUUGAAGGGAGCUCUG |
| 74 | UUUGGUUUGAAGGGGGCUCUG |
| 75 | UUGGACUGAAGGGUGCUCCCU |
| 76 | CUGCACUGCCUCUUCCCUGGC |
| 77 | UGCAGUUGCUGCCUCAAGCUU |
| 78 | UGCAGUUGUUGUCUCAAGCUU |
| 79 | UGCAUUUGCACCUGCACCUAC |
| 80 | UUAGAUGACCAUCAGCAAACA |
| 81 | UUUCCAAAUGUAGACAAAGCA |
| 82 | UCGCUUGGUGCAGGUCGGGAC |
| 83 | UGCACUGCCUCUUCCCUGGCU |
| 84 | UUGACAGAAGAUAGAGGGCAC |
| 85 | UUCCACGGCUUUCUUGAACUG |
| 86 | UCGGACCAGGCUUCAUUCCCG |
| 87 | UAACCUGGCUCUGAUACCA |
| 88 | UCGGACCAGGCUUCAUUCCCU |
| 89 | UUAGAUGACCAUCAACAAACA |
| 90 | CAGCCAAGGAUGAUUUGCCGG |
| 91 | UCUCAUUCCAUACAUCGUCUGA |
| 92 | UCUCAUUCCAUACAUCGUCUG |
| 93 | UAGAAAGGGGAAUAGCAGUUG |
| 94 | UAGAAAGGGAAAUAGCAGUUG |
| 95 | UUAAUCAAGGAAAUCACGGUCG |
| 96 | UUAAUCAAGGAAAUCACGGUU |
| 97 | AGGGAUAGGUAAAACAACUACU |
| 98 | UUGACAGAAGAGAGUGAGCAC |
| 99 | GCGUAUGAGGAGCCAAGCAUA |
| 100 | UCUCGGACCAGGCUUCAUUC |
| 101 | UCUUCCCUACACCUCCCAUACC |
| 102 | GGACAGUCUCAGGUAGACA |
| 103 | UUGUUCGAUAAAACUGUUGUG |
| 104 | UCAGAUCAUCUUGCAGCUUCA |
| 105 | UUGCCGAUUCCACCCAUUCCUA |
| 106 | UGAGACCAAAUGAGCAGCUGA |
| 107 | AGCCGUAAACGAUGGAUACU |
| 108 | UGGAGAAGCAGGGCACGUGAA |
| 109 | UGCAGUUGCUGUCUCAAGCUU |
| 110 | CAGGGAAGAGGCAGAGCAUGG |
| 111 | CUGACAGAAGAUAGAGAGCAC |
| 112 | UUGAGUGCAGCGUUGAUGAAA |
| 113 | UGACGACGAGAGAGAGCACGC |
| 114 | UUGGACCAGGCUUCAUUCCCC |
| 115 | UCUUCCCUACUCCACCCAUUCC |
| 116 | GGAAUGUUGGCUGGCUCGAGG |
| 117 | UUACCGAUUCCACCCAUUCCUA |
| 118 | ACAGGGAACAUGCAGAGCAUG |
| 119 | UGACAGAAGAGAAUGAGCAC |
| 120 | UUUCCAAUUCCACCCAUUCCUA |
| 121 | UUGAUACGCACCUGAAUCGGC |
| 122 | UAGGAUUCAAUCCUUGCUGCU |
| 123 | GCUCACUUCUCUUUCUGUCAGC |
| 124 | UGGAGAAGCAGGGCACGUGAG |
| 125 | UCGAACCAGGCUUCAUUCCCC |
| 126 | GGAUUGUUGUCUGGUUCAAGG |
| 127 | AGGUCAUGCUGUAGUUUCAUC |
| 128 | CGAUCUUGAGGCAGGAACUGAG |
| 129 | CGGUCUUGAGGCAGGAACUGAG |
| 130 | CUAGAUUUGUUUAUUUUGGGACGG |
| 131 | CAGGGAUGAGGCAGAGCAUGG |
| 132 | UCAUCCUCAUCAUCAUCGUCC |
| 133 | UGUUGUCUCAAGCUUGCUGCC |
| 134 | UGCAGUUGUUGCCUCAAGCUU |
| 135 | UGAGGAAUCACUAGUAGUCGU |
| 136 | UAAACAGUGCCCACCCUUCAUC |
| 137 | GGUCUAGGUGGAGUUGGAAAAA |
| 138 | UUCCACGGCUUUCUUGAACUU |
| 139 | UCUUCCCUACUCCUCCCAUUCC |
| 140 | UCUUCCCUAUUCCUCCCAUUCC |
| 141 | UCUUUCCUACUCCUCCCAUUCC |
| 142 | UAUCGGAAUCUGUUACUGUUUC |
| 143 | UCACAUCUGGGCCACGAUGGUU |
| 144 | UGUUCUUGACGUCUGGACCACG |
| 145 | UUUGGAGAGAAAAUGGCGACAU |
| 146 | CCGGCCUCGAAUGUUAGGAGAA |
| 147 | CGUGGGACAGCAUAGAAUGCG |
| 148 | UCAUCCUCAUCAUCCUCGUCC |
| 149 | CGUUUCACGUCGGGUUCACC |
| 150 | UCGGCCUUGAAUGUUAGGAGAA |
| 151 | AGAAUCCUGAUGAUGCUGCAA |
| 152 | UUUUCCCUACUCCACCCAUCCC |
| 153 | UUUUCCCAACUCCACCCAUCCC |
| 154 | CCGACCUUAGCUCAGUUGGUG |
| 155 | AAACCUGGCUCUGAUACCA |
| 156 | UGACAGAAGAGAGCGAGCAC |
| 157 | CCUGCCUUGCAUCAACUGAAU |
| 158 | UCUUGCCUACACCGCCCAUGCC |
| 159 | UGCACUGCCUCUUCCCUGGCUG |
| 160 | UUGAACAUCCCAGAGCCACCG |
| 161 | UGAACAUCCCAGAGCCACCGG |
| 162 | UCUGGCGAGGGACAUACACUGU |
| 163 | UGACAGAGGAGAGUGAGCAC |
| 164 | GCUCACUUCUCUCUCUGUCAGU |
| 165 | CCCGCCUUGCAUCAAGUGAA |
| 166 | CAGGGACGAGGCAGAGCAUGG |
