# Supplementary material for: Prediction of plant-derived xenomiRs from plant miRNA sequences using random forest and one-dimensional convolutional neural network models
Source: BMC Genomics. 2018 Nov 26;19:839. doi: 10.1186/s12864-018-5227-3 (PMC6258294; doi:10.1186/s12864-018-5227-3)
Supplement: Supplementary file 2 — Table S2. Negative samples. (DOCX 50 kb) [file 12864_2018_5227_MOESM2_ESM.docx]

| **No.** | **Sequence** |
| --- | --- |
| 1 | AAAAAACAAGGAUCCACGGAU |
| 2 | AAAACAUCUGAUAACCUGAAACGG |
| 3 | AAAACGGAGCGGUCCAUUAGCGCG |
| 4 | AAAAGGAUCAUACCGUGACAG |
| 5 | AAACGAACAAAAAACUGAUGG |
| 6 | AAAGAUCUUUAGUCCCGGUUGUUC |
| 7 | AAAGAUGUGCAUGUAGUUCCG |
| 8 | AAAGGUAGAGUCAGGUAUGAG |
| 9 | AAAGUAUAAUGGUUUAGUGGUUUG |
| 10 | AAAGUUAGAGAAGUUUGACUU |
| 11 | AAAUGACCAAAAUACCCCUAGAAC |
| 12 | AAAUUACUUGUCGUUCUAGCU |
| 13 | AACAAUAGGAAUGGGAGGCAU |
| 14 | AACCAGGCUCUGAUACCAUG |
| 15 | AACCGCUGUUUAGGCGGAGUGG |
| 16 | AACCGGCAUCUGUAAUAUAUUAUA |
| 17 | AACCUAGUACUGGAUUAGUCACCA |
| 18 | AACCUAUUUAACGACAUGACU |
| 19 | AACUAGAGUGGGUCAACGGGUACC |
| 20 | AACUCUGAUUCUAGAAUUUUUG |
| 21 | AACUUUGUGAUGACAACGAAG |
| 22 | AACUUUUUAAUAGCUACAACUUCU |
| 23 | AAGAAAACGGAUGCGCGUGCUA |
| 24 | AAGAACAUCAAAUUAGAAUGU |
| 25 | AAGAACUUAAUAUAACUUUAAAGC |
| 26 | AAGACGAAUAAUCAAACGUUGGAC |
| 27 | AAGACGAUCCUAGCAGAGCUUCAU |
| 28 | AAGACGGACAAUCAAACGUUGGAC |
| 29 | AAGACGGAUGAUUAAAGUUGGACA |
| 30 | AAGACGGUUUUAGAUGUUGCC |
| 31 | AAGAUAAGCGCCUUAGUUCUG |
| 32 | AAGCAGCGACUCUGGUCAUGGA |
| 33 | AAGCCAAGGAUGACUUGCCGG |
| 34 | AAGCUCAGGAGGGAUAGCACC |
| 35 | AAGGAAUUGGGUCUAAUCUCUUCC |
| 36 | AAGGAGUGGCAUGUGAACACA |
| 37 | AAGGGGAUUGAGGAGAUUGGG |
| 38 | AAGGGGGGGGGGGGAAAGA |
| 39 | AAGUAGACACAUAAGAAGGAG |
| 40 | AAGUCAUCAACAAAAAAGUUGAAU |
| 41 | AAGUGCAGCCAUGAGUUUCCGUGC |
| 42 | AAGUUUGGACUUAAAUUUGGUAAC |
| 43 | AAUCAAGUUAGGAACCAUGCAAGU |
| 44 | AAUCCCUUAUAUUAUGGGACGG |
| 45 | AAUCGACGGCCUCAGUCAGGG |
| 46 | AAUGAGAAUGAUGACCCGGUG |
| 47 | AAUGCAGUUUGAUCCAAGAUC |
| 48 | AAUGCGCAACUCUAUAUUUCC |
| 49 | AAUGGAGGCAAGUGUGAAGGA |
| 50 | AAUGGUAGUGACAUUAUGGUAG |
| 51 | AAUGUUAUGGACACUAGAUGACAU |
| 52 | AAUGUUAUUGUAGCGUGGUGGUGU |
| 53 | AAUGUUUGUAUGGAUCGUUUGU |
| 54 | AAUUCACAGGCCCUAUCUUGUG |
| 55 | AAUUGACAGUAUUAUGCCAGA |
| 56 | AAUUGCUGGCAGCACUGUUUA |
| 57 | AAUUGGGUUUAUGCUAGAGUU |
| 58 | ACAAAAUCCGUCUUUGAAGA |
| 59 | ACAAAGUUUUAUACUGACAAU |
| 60 | ACAACUCUUGUGGAUGGAGGG |
| 61 | ACAAUGGAGUGAAGUGCAACAGAA |
| 62 | ACACUAAAGGACCUAAACUAAC |
| 63 | ACACUGAAGGACCUAAACUAAC |
| 64 | ACACUUAGUUUUGUACAACAU |
| 65 | ACAGAAGAUAGAGAGCACAG |
| 66 | ACAGGCAAAGACAAUGACGGC |
| 67 | ACAGUGGUCAUCUGGUGGGCU |
| 68 | ACAGUUUGUGUUUUGUUUUGU |
| 69 | ACAUAUGAUCUGCAUCUUUGC |
| 70 | ACAUCUUCCAAUUAAUGCAU |
| 71 | ACAUGCUGGAUCUACUUGAAG |
| 72 | ACAUGUUUACAAGUUAAAAUGU |
| 73 | ACAUUAUGGGACGGAGGGAGU |
| 74 | ACCAAAUCGUUUUCGAUCGUUGGA |
| 75 | ACCACUGUAGACGCUACGUGUGAG |
| 76 | ACCAGCAUUCCAUUGGCAGAGG |
| 77 | ACCGGAUGUCGGAAAAGGUUU |
| 78 | ACCGGUUGGAACUUGCCUUAA |
| 79 | ACCUGUGACGGGCCGAGAAUGGAA |
| 80 | ACCUGUUUGGAUUCUUGAGGGCUA |
| 81 | ACGAAACCAUAGGGAUUUUGGCAU |
| 82 | ACGAACGGUUAAAGUUGGACGCGG |
| 83 | ACGAGGUUGGUUUAUUUUGGGACG |
| 84 | ACGAGUGAUGGCGGCGUAUACCUG |
| 85 | ACGGAAAAUCAUGGCUGCACUUAA |
| 86 | ACGGAACCCUAAUGGCGAUGGCAU |
| 87 | ACGGAUGAUUAAAGUUGGACACGG |
| 88 | ACGGAUGUAGAAAUUGGUGAU |
| 89 | ACGGUAUCCCGUUCGGACAGGAUG |
| 90 | ACGGUAUCUCUCCUACGUAGC |
| 91 | ACGUGAUGUGCCACGUAGGCA |
| 92 | ACGUUAGACACGAAUUUCUACGGC |
| 93 | ACGUUGGGUACGAAUAUCUACGGC |
| 94 | ACUAAAUCGUUUCUGAUCGUUGGA |
| 95 | ACUAGGUUUGUUUAUUUUGGGACG |
| 96 | ACUCAUAAGAUCGUGACACGU |
| 97 | ACUGAACUCAAUCACUUGCUGC |
| 98 | ACUGAAGUAGAGAUUGGGUUU |
| 99 | ACUGUAUAUAUGUAAGUGACA |
| 100 | ACUUAAUCUGGACACUAUAAAAGA |
| 101 | ACUUGGCUGAUUCUAUUAUU |
| 102 | ACUUUAAUACCAUUGGAAGAUUGC |
| 103 | ACUUUGAAGCUUUGAUUUGAA |
| 104 | AGAAAACCAGCUUCCAGAUCU |
| 105 | AGAAGAGAGAGAGUACAGCUU |
| 106 | AGAAGCAAAAUGACGACUCGG |
| 107 | AGAAGCGUACAAGGAGAUGAGG |
| 108 | AGAAGCUGCAGCUGUCAGAAGCUC |
| 109 | AGAAGGACUAGUAAGAUGGCU |
| 110 | AGAAGGAGAAUAGAUAUGGUU |
| 111 | AGAAGUUGUGGCUAUCAAAAAGUU |
| 112 | AGAAUCUUGAUGAUGAUUAAA |
| 113 | AGAAUCUUGAUGAUGCUGCA |
| 114 | AGAAUUGGAGGAAAACAAACUGA |
| 115 | AGACAAAAAUAUAAAUAACAAA |
| 116 | AGACCAUGCCUAUACCUACCA |
| 117 | AGACUACAAUUAUCUGAUCA |
| 118 | AGAGAGUGUAGAAAGUUUCUCA |
| 119 | AGAGAUGGGACGGGCAGGGAAG |
| 120 | AGAGGUCCGGAUCGAACGUAG |
| 121 | AGAGGUGACCAUUGGAGAUG |
| 122 | AGAGUUGGAGGAAAGAAAACU |
| 123 | AGAGUUGGAGGAAAGCAAACC |
| 124 | AGAUAUUAGUGCGGUUCAAUC |
| 125 | AGAUAUUGGUACGGUUCAAUC |
| 126 | AGAUAUUGGUGCGGUUCAAUC |
| 127 | AGAUGACAUGUGAAUGAUGAGGGG |
| 128 | AGAUGUGGAGAUCGUGGGGAUG |
| 129 | AGAUUGCUUUCAAGGUCAUUUCUU |
| 130 | AGCAAAAGCUAAGGAAAAGGAA |
| 131 | AGCAACGAUUUUAAGAUUGUGGCA |
| 132 | AGCACCAGUUGACGUGACGCUGAG |
| 133 | AGCACUGGAGUAGCCAAGAGA |
| 134 | AGCAUUCAAACAUUCCCAAUUACC |
| 135 | AGCCAAGGAUGACUUGCCGG |
| 136 | AGCCGGGCUCGGUCGCGCGUG |
| 137 | AGCCUGUAAAACUGCAAAAAGGAA |
| 138 | AGCGCCCAAGCGGUAGUUGUC |
| 139 | AGCUAAGGAUUUGCAUUCUCA |
| 140 | AGCUCAGGAGGGAUAGCGCC |
| 141 | AGCUCUGAUACCAUGUUAACUGUU |
| 142 | AGCUCUGAUACCAUGUUAGAUUAG |
| 143 | AGCUGCUGACUCGUUGGCUC |
| 144 | AGCUGCUUAGCUAUGGAUCCC |
| 145 | AGCUUCUGACAGCUGCAGUUUCUC |
| 146 | AGGACGAGGGGAACGGCGGCG |
| 147 | AGGAGGUCUGGAGAGUUAUGUGGC |
| 148 | AGGAUUAGAGGCAACUGAACC |
| 149 | AGGCAACUACACGUUGGGCGCUCG |
| 150 | AGGCAGCUCUCCUCUGGCAGG |
| 151 | AGGCUGAUCCACUCCAGAGGA |
| 152 | AGGCUUGGUGCAGCUCGGGAA |
| 153 | AGGCUUUUAAGAUCUGGUUGC |
| 154 | AGGGACUUUGUGAAUUUAGGG |
| 155 | AGGGGCUUGCAUAUUCUACCA |
| 156 | AGGGUUGAUAUGAGAACACAC |
| 157 | AGGUAUUGGCGUGCCUCAAUC |
| 158 | AGGUCAUGCUGGUAGUUUCAC |
| 159 | AGGUGCAGAGGCAGAUGCAAC |
| 160 | AGGUGCUGAGUGUGCUAGUGC |
| 161 | AGGUGGGCAUACUGCCAAUAG |
| 162 | AGGUGGGCAUACUGCCAAUG |
| 163 | AGGUGGGCAUACUGUCAACU |
| 164 | AGGUGGGUAUACUGCCAAUA |
| 165 | AGUAUAGGUGUCGGCUCUAUU |
| 166 | AGUAUGCCCACUACCUAUC |
| 167 | AGUAUUAGGAUACGUCUCAUC |
| 168 | AGUCUGAUAAGAAGUCAAUGGCGU |
| 169 | AGUGACGGGAGGACUGCAAGG |
| 170 | AGUGAGGAGGCCGGGGCCGCU |
| 171 | AGUGGCUGCAUAUCUGAUGAG |
| 172 | AGUGGUGUCGUAUUACCUGCC |
| 173 | AGUGUCUCCUGAUGAUCGGGACAA |
| 174 | AGUUGCCUACAUGGAGCGCCA |
| 175 | AUAAAUCCCAACAUCUUCCA |
| 176 | AUAAGAGCUGUUGAAGGAGUC |
| 177 | AUAAGUGGGUUUGUGGGCUGGCCC |
| 178 | AUACCAAAACUCUCUCACUUU |
| 179 | AUACUCAAACAGGGCAUUGCA |
| 180 | AUAGACAGAGUCACUCACAGA |
| 181 | AUAGCAAAUCGAUAAGCAAUG |
| 182 | AUAGCCUUGAACGCCGUCGUU |
| 183 | AUAGGUGGGCCAGUGGUAGGA |
| 184 | AUAGUUCAAGAAAGUCCUUGGAAA |
| 185 | AUAUGCUGGAUCUACUUGAAG |
| 186 | AUAUGGAUUCAGAAUGCAGGU |
| 187 | AUCACAAUACAAUAAAUCUGGA |
| 188 | AUCAGGACCAGUAGGCGAUGGUAA |
| 189 | AUCAGGAGAGAUGACACCGAC |
| 190 | AUCAGUGCAAUCCCUUUGGAAU |
| 191 | AUCAGUUUCUUGUUCGUUUCA |
| 192 | AUCAUGCAUGACAGCCUCAUUU |
| 193 | AUCCUCGGGAUACAGUUUACC |
| 194 | AUCGAAUUUGAAAGAAAAAGGUAU |
| 195 | AUCUGAAGAAAAUAGCGGCAU |
| 196 | AUCUGGAAGCUAGGUUUUCUCU |
| 197 | AUGAAGUGUUUGGAGGAACUC |
| 198 | AUGAAUUUGGAUCUAAUUGAG |
| 199 | AUGACAAGGCCAAGAUAUAACA |
| 200 | AUGAGGUUGGUUUAUUUUGG |
| 201 | AUGAGUUGGGUCUAACCCAUAACU |
| 202 | AUGAUGAUGAUGAUGAUGAAA |
| 203 | AUGGAAUAUAUGACAAAGGUGG |
| 204 | AUGGACUGUACUUUGUAAAGCGGA |
| 205 | AUGGAUCAUCUAGAUGGAGAU |
| 206 | AUGGCAUGAAAGAAGGUGAGA |
| 207 | AUGGCUCUGAUAUCAUGUUGGUUU |
| 208 | AUGGCUGCACUUAAAAUGGGACGG |
| 209 | AUGGUUCAAGAAAGCCCAUGGAAA |
| 210 | AUGUAGAUGAGGUGAUGUUACACA |
| 211 | AUGUCGAGGUCGAGUUCCCGGC |
| 212 | AUGUGAACAGUGUCAAACAGUGUC |
| 213 | AUGUGCCCAUCUUCUCCACC |
| 214 | AUGUGCUGAUUCGAGGUGGGA |
| 215 | AUGUGUAUAGGGAAGCUAAUC |
| 216 | AUUAACGCUGGCGGUUGCGGCAGC |
| 217 | AUUCAAGAUAGCUGUGGAAAA |
| 218 | AUUCAUAGUACUAGAUGUGU |
| 219 | AUUCGUUGUAUUAAGAGAGGGUU |
| 220 | AUUGAGUGCAGCGUUGAUGA |
| 221 | AUUGGAUUGAAGGGAGCUCCG |
| 222 | AUUGGAUUGAAGGGAGCUCCU |
| 223 | AUUGGGAUUCAGUUGGAGUUGG |
| 224 | AUUGGUAGUGGAUAAGGGGGC |
| 225 | AUUGGUUUGAAGGGAGCUCCA |
| 226 | AUUUAACUCAAGUGAGCAUUGU |
| 227 | AUUUACAGUCAUAUACAGUUUAUA |
| 228 | AUUUCUAGUGGGUCGUAUUCA |
| 229 | AUUUGAGUCAUGUCGUUAAG |
| 230 | AUUUGUACACCUAGAUCUGUA |
| 231 | AUUUGUUACUAAUUUGGAAUG |
| 232 | CAAAUGAAGUUGAGUUUGGAC |
| 233 | CAACGAGGAGGCCGGGACCA |
| 234 | CAACUGAAGUCGGUGUUUACU |
| 235 | CAACUGAGCUUCCUCAUCUCU |
| 236 | CAACUUAAAAUCAGUUGGCAAU |
| 237 | CAAUUUCUAGUGGGUCGUAUU |
| 238 | CACGUGCGCUCCUUCUCCAAC |
| 239 | CACGUGCUCCCCUUCUCCACC |
| 240 | CACGUGGUCUCCUUCUCCAU |
| 241 | CACGUGUUCUACUCCUCCAAC |
| 242 | CACGUGUUCUCCUUCUCCAUC |
| 243 | CACUUCAUAGUACAACGAAUCU |
| 244 | CAGAGCUGUGGCAUCCACGUCG |
| 245 | CAGAUAGAGAAUCUUCUCAGA |
| 246 | CAGCAAGAACUGGAUCUUAAU |
| 247 | CAGCACCACCAAGAUUCACA |
| 248 | CAGCACCAUCAAGAUUCACA |
| 249 | CAGCCAAGAAUGACUUGCCGG |
| 250 | CAGCCAAGGAUGACUUGCCGU |
| 251 | CAGCCAAGGGUGAUUUGCCGG |
| 252 | CAGCUCCUGCAGCAUCUGUUC |
| 253 | CAGGAUUUUAUACAUGUAAAGAAU |
| 254 | CAGGUGUCAUCUCCCCUGAAC |
| 255 | CAGGUGUUCUCGAUGGCUUCC |
| 256 | CAGUCUUGUGGCAAGAACUGAG |
| 257 | CAUAUCCCAGCUGUUUCGGCC |
| 258 | CAUCCAAGGUGUUUGUAGAAA |
| 259 | CAUGGAAGUGAAUCGGGUGAC |
| 260 | CAUGGUGCCGGUUCCGGUGGCG |
| 261 | CAUGUGCCCUUCUUCUCCAUC |
| 262 | CAUUCAAGGACUUCUAUUCAG |
| 263 | CCAGCCGGUGGCAUAGUUCUC |
| 264 | CCAGCGCUGCACUCAAUUACG |
| 265 | CCAGGACGUGUGGGAUGGCA |
| 266 | CCAUAUUCGUAGUGCUAGGUGG |
| 267 | CCAUCUUACUAGUCUUUCUUU |
| 268 | CCCCUUACAAUGUCGAGUAAA |
| 269 | CCCGGCUAUCGGAACGGCUGC |
| 270 | CCGAGGUUUUCUGGAUACAUU |
| 271 | CCGAUGGUUGUUCUACAGGUG |
| 272 | CCGUAUCUUGGCCUUGUCAUU |
| 273 | CCUAACCUAGGAGUUCGAUGGGAC |
| 274 | CCUCAUUCCAAACAUCAUCU |
| 275 | CCUCGCCGGCGCGCGCGUGCA |
| 276 | CCUCGUUCGACCGUGGCAUUU |
| 277 | CCUGAGGGGAAAUCGGCGGGA |
| 278 | CCUUCUCAUCGAUGGUCUAGA |
| 279 | CCUUGGAGAAAUAUGCGUCAA |
| 280 | CCUUUAAUAUAUUAUAGGUGUCGG |
| 281 | CGAAGAAUCGCAGUCACUAGUUGU |
| 282 | CGACAGAAGAGAGUGAGCAUA |
| 283 | CGAGAUCGAUGGUAUAUUUCUG |
| 284 | CGAGGACGAGAUACAGUGCAG |
| 285 | CGAUCUUGUAGCAAGAACUGAG |
| 286 | CGAUCUUGUGGCAGGAGCUGAG |
| 287 | CGAUGAAGGUCUUUGGAACGGUA |
| 288 | CGAUGACAACAGCUAGAAUGG |
| 289 | CGAUGUUGGCAUGGCUCAAUC |
| 290 | CGAUUCCCCAGCGGAGUCGCCA |
| 291 | CGCAAAUGCGGAUAUCAAUGU |
| 292 | CGCUAUCCAUCCUGAGUUUC |
| 293 | CGCUAUCCAUCCUGAGUUUCA |
| 294 | CGCUAUCUAUCCUGAGCUCC |
| 295 | CGCUAUCUAUCCUGAGCUCCA |
| 296 | CGGACCAGGCUUCAUUCCCC |
| 297 | CGGCUCUGAUACCAAUUGAUG |
| 298 | CGGUCUUGUGGCAAGAACUGAG |
| 299 | CGGUGGAUCUUGUUUUUUGU |
| 300 | CGGUUUGUCAAGCGGAGUGC |
| 301 | CGUACAAGGAGUCAAGCAUGA |
| 302 | CGUCUGGGAUGGCAUUUUGGC |
| 303 | CGUGAAGAGCUCGCCGGCGGC |
| 304 | CGUGACGAACAUAAUCAAACG |
| 305 | CGUGUUCAACGUUUGACUGUC |
| 306 | CGUUUUGCAAAUUCGCAGGCC |
| 307 | CUAGAACGGCAAGCAAUUUGA |
| 308 | CUCAAGAAAGCUGUGGGAGA |
| 309 | CUCAAGGGUGUUUGUGAAAUA |
| 310 | CUCAGCAGUCGACUGUACCGUG |
| 311 | CUCAGGAGAGAUGACACCGAC |
| 312 | CUCAUUGCUUCGGUAGGCUGG |
| 313 | CUCCUGAUGCCUCCCAAGCCUA |
| 314 | CUCGCCGGGGCUGCGUGCCG |
| 315 | CUCGCCGGGGCUGCGUGCCGCCAU |
| 316 | CUCGGAAGCUAGACGUGUGGCAGG |
| 317 | CUCGGACCAGGCUUCAUUCCC |
| 318 | CUGAAACUGAGACUGCAUCUGG |
| 319 | CUGAUAAUUUUACGAUGAAUAG |
| 320 | CUGGACUACAAGAUCCCGGAU |
| 321 | CUGGCCGAGGCCGUCGAUUCU |
| 322 | CUGGGAACAGGCAGGGCACG |
| 323 | CUGGGUGAGAGAAACACGUAU |
| 324 | CUGUACCCUCUCUCUUCUUC |
| 325 | CUUCCAUAUCUGGGGAGCUUC |
| 326 | CUUCCAUUUAUGAUAAGUAU |
| 327 | CUUCGGGGGAGGAGAGAAGC |
| 328 | CUUCUUAAGUGCUGAUAAUGC |
| 329 | CUUGGACUGAAGGGAGCUCCC |
| 330 | CUUGGAUUGAAGGGAGCUCCU |
| 331 | CUUGGAUUGAAGGGAGCUCUA |
| 332 | CUUGUUUGUGGUGAUGUCUAG |
| 333 | CUUGUUUUUCUCCAAUAUCUCA |
| 334 | CUUUAUAUCCGCAUUUGCGCA |
| 335 | CUUUAUUGUUGAUGUCAAAA |
| 336 | CUUUGCCAGAAGCCCUCAUGG |
| 337 | GAAAGACCAAACGAGAAGCUGCAU |
| 338 | GAAAUAGCGAAGAUAUGAUUA |
| 339 | GAAAUCGGAGAGGAAAUUCGCC |
| 340 | GAAAUGAUCUUGGACGUAAUCUAG |
| 341 | GAAAUGGGAGCAGAGCAGGUUU |
| 342 | GAACGGUCAAAUGUUAGACACGGA |
| 343 | GAACUGUAAGUCUGUGACGGGUAA |
| 344 | GAAGGAAGAAUCGUUAUGGAA |
| 345 | GAAGGCCACCGUCGGGAUCGC |
| 346 | GAAUGACGUCCGGUCUGAAGA |
| 347 | GAAUGGAGGCUGGUCCAAGA |
| 348 | GAAUGUAGUGAAUUUGUUCCA |
| 349 | GAAUGUUGUCUGGAUCGAGG |
| 350 | GAAUGUUGUUUGGAUCGAGG |
| 351 | GACCGUAGAAACUAGCAUAGAAAA |
| 352 | GACGGACGAUUAAAGUUGGGCAUG |
| 353 | GACGGACGGUUAAACGUUGGAC |
| 354 | GACGUCCAUCGAUGAAGAGCGA |
| 355 | GAGAGGAGGACGGAGUGGGGC |
| 356 | GAGCCAAGGAUGACUUGCCGG |
| 357 | GAGCUCCCUUCGAUCCAAUCC |
| 358 | GAGCUCCUAUCAUUCCAAUGA |
| 359 | GAGCUCCUCUCAUUCCAAUGA |
| 360 | GAGCUCCUUGAAGUCCAAUU |
| 361 | GAGCUCCUUGAAGUCCAAUUG |
| 362 | GAGGAAAGUGGGCAGUUGGGUU |
| 363 | GAGGAGCUGUGACGAUUUGGGA |
| 364 | GAGGAUUCGGUAUUGAUCGCUA |
| 365 | GAGGGAUUUUGCGGGAAUUUCACG |
| 366 | GAGGUGAGCCGAGCCAAUAUC |
| 367 | GAGGUGUUUGGGAUGAGAGAA |
| 368 | GAUAUUGGCGCGGUUCAAUC |
| 369 | GAUCAUGCUGUGACAGUUUCACU |
| 370 | GAUCAUGCUGUGCAGUUUCAUC |
| 371 | GAUCAUGCUGUGGCAGCCUCACU |
| 372 | GAUCAUGUGGCAGUUUCAUU |
| 373 | GAUCAUGUUGCAGCUUCAC |
| 374 | GAUCCAAAAAAGUGCCACGUGAGC |
| 375 | GAUCGUGCUGCGCAGUUUCACC |
| 376 | GAUGACAGCGGUGGUUCGGACAUC |
| 377 | GAUGAGGAUAGGGAGGAGGAG |
| 378 | GAUGGAUAUGUCUUCAAGGAC |
| 379 | GAUUCUCUGUGCAAGUGGAAG |
| 380 | GCAACAUCUUCAAGAUUCAGA |
| 381 | GCAAGUUGACCUUGGCUCUGC |
| 382 | GCAAGUUGACCUUGGCUCUGU |
| 383 | GCAGCACCAUCAAGAUUCAC |
| 384 | GCAGCACCAUUAAGAUUCAC |
| 385 | GCAUUGAGGGAGUCAUGCAGG |
| 386 | GCCAGAUGUGUUAAAAUAAUGACC |
| 387 | GCGCUCCACGUAGGCAACAAU |
| 388 | GCGGCGAGGGGAUGCGAGCGUG |
| 389 | GCGUACAAGGAGCCAAGCAUG |
| 390 | GCGUACAGAGUAGUCAAGCAUG |
| 391 | GCGUGCAAGGAGCCAAGCAUG |
| 392 | GCGUGCAAGGGGCCAAGCAUG |
| 393 | GCGUGCACGGAGCCAAGCAUA |
| 394 | GCGUGCAUGGUGCCAAGCAUA |
| 395 | GCGUGCGAGGAGCCAAGCAUG |
| 396 | GCGUGCGAGGUGCCAAGCAUG |
| 397 | GCGUGCGAGGUGCCAGGCAUG |
| 398 | GCGUGCGUGGAGCCAAGCAUG |
| 399 | GCUAGAGGUGGCAACUGCAUA |
| 400 | GCUCAAGAAAGCUGUGGGAGA |
| 401 | GCUCACCCUCUAUCUGUCAGU |
| 402 | GCUCACUCUCUAUCUGUCAGC |
| 403 | GCUCACUGCUCUAUCUGUCACC |
| 404 | GCUCACUGCUCUAUCUGUCAUC |
| 405 | GCUCACUGCUCUCUCUGUCAUC |
| 406 | GCUCACUGCUCUUUCUGUCAUC |
| 407 | GCUCGCUCCUCUUUCUGUCAGC |
| 408 | GCUCUCUAAGCUUCUGUCAUC |
| 409 | GCUGAUUCUCUGAUUUUGAAC |
| 410 | GCUUACUCUCUAUCUGUCACC |
| 411 | GGAAUCUUGAUGAUGCUGCAU |
| 412 | GGAAUGACGUCCGGUCCGAAC |
| 413 | GGAAUGGGCUGAUUGGGAAGCA |
| 414 | GGAAUGUCGUCUGGCGCGAGA |
| 415 | GGAAUGUUGUCUGGCACGAGG |
| 416 | GGAAUGUUGUCUGGCUCGGGG |
| 417 | GGAAUGUUGUCUGGUCCGAG |
| 418 | GGAAUGUUGUCUGGUUGGAGA |
| 419 | GGAAUGUUGUUUGGCUCGAGG |
| 420 | GGACCAGGCUUCAUUCCCC |
| 421 | GGACUGUUGUCUGGCUCGAGG |
| 422 | GGAGAUGGGAGGGUCGGUAAAG |
| 423 | GGAGAUUCUUUCAGUCCAGUC |
| 424 | GGAGCAUCAUCAAGAUUCACA |
| 425 | GGAGGCAGCGGUUCAUCGAUC |
| 426 | GGAGGCGUAGAUACUCACACC |
| 427 | GGAUAUUGGUGCGGUUCAAUC |
| 428 | GGAUUGAGCCGCGUCAAUAUC |
| 429 | GGCAAGUCAUCUGGGGCUACG |
| 430 | GGCAAGUCUGUCCUUGGCUACA |
| 431 | GGCAAGUUGUCCUUGGCUAC |
| 432 | GGCAAGUUGUCCUUGGCUACA |
| 433 | GGCAAGUUGUUCUUGGCUACA |
| 434 | GGCAAUAACUUGAGCAAACA |
| 435 | GGCAGCCGAGCGAGGGCCUCGG |
| 436 | GGCAGGCCUUCUGGCUAAG |
| 437 | GGCAGUCUCCUUGGAUAUC |
| 438 | GGCAGUCUCCUUGGCUAG |
| 439 | GGCAGUCUCCUUGGCUAUC |
| 440 | GGCAGUCUCCUUGGCUAUU |
| 441 | GGCAGUCUCUUUGGCUAUC |
| 442 | GGCAGUCUUCUUGGCUAUC |
| 443 | GGCAUCCAUUCUUGGCUAAG |
| 444 | GGCAUGUCUUCCUUGGCUACU |
| 445 | GGCCGGUGGUCGCGAGAGGGA |
| 446 | GGCUGUACAAAAGGAAACUAC |
| 447 | GGGAGCAUGUAGGAUGGCCAU |
| 448 | GGGAGGAAACAGUGCCUAGUG |
| 449 | GGGCAACUUCUCCUUUGGCAGA |
| 450 | GGGCAAGAUCACCAUUGGCAGA |
| 451 | GGGCAAGCCGCCGCCGCCAC |
| 452 | GGGCAUCUUUCUAUUGGCAGG |
| 453 | GGGCGCAGUGGUUUAUCGAUC |
| 454 | GGGCUUCUCUUUCUUGGCAGG |
| 455 | GGGGAUAGAUCGACGCGUCAAG |
| 456 | GGGGCGAACUGAGAACACAUG |
| 457 | GGGGCGGACUGGGAACACAUG |
| 458 | GGGUAAGAUCUCUAUUGGCAGG |
| 459 | GGGUACGUCUCCUUUGGCACA |
| 460 | GGGUGUCAUCUCGCCUGAAGCA |
| 461 | GGUACCCUUUCAGAUAGUCUCA |
| 462 | GGUCAAGAAAGCCGUGGGAAG |
| 463 | GGUCAAGAAAGCUGUGGGAAG |
| 464 | GGUCAUGCUGCGGCAGCCUCACU |
| 465 | GGUCAUGCUGCUGCAGCCUCACU |
| 466 | GGUCAUGCUGUAGUUUCAUC |
| 467 | GGUUCGUACGUACACUGUUCA |
| 468 | GUAAUAUACUAAUCCGUGCAU |
| 469 | GUAGCAUCAUCAAGAUUCAC |
| 470 | GUAGCAUCAUCAAGAUUCACA |
| 471 | GUAGGGAUAGGCAUGAUCUCU |
| 472 | GUAGUACUCGGUUGUAGGUGUA |
| 473 | GUCAGUGCAAUCCCUUUGGAAU |
| 474 | GUCAUGGGGUAUGAUCGAAUG |
| 475 | GUCCUCGGGAUGCGGAUUACC |
| 476 | GUCCUUGGGAUGCAGAUUACG |
| 477 | GUCGUUGUAGUAUAGUGG |
| 478 | GUGAAGCGUUUGGGGGAAAUC |
| 479 | GUGAAGUAUUUGGCGGAACUC |
| 480 | GUGAAGUGCUUGGGGGAACUC |
| 481 | GUGAAGUGUUUGAGGAAACUC |
| 482 | GUGAAGUGUUUGGAGGAACUC |
| 483 | GUGAAGUGUUUGGGGAAACUC |
| 484 | GUGAAGUGUUUGGGGGAACUC |
| 485 | GUGAAGUGUUUGGGGGAUUCUC |
| 486 | GUGAAGUGUUUGGGUGAACUC |
| 487 | GUGAAUUGUUUGGGGGAACUC |
| 488 | GUGACAUAUUUUACUACAAC |
| 489 | GUGAGCCGAACCAAUAUCACU |
| 490 | GUGCAGCUCUCCUCUGGCAUG |
| 491 | GUGCAGUUCUCCUCUGGCACG |
| 492 | GUGCAUUAAUUGGAAGAACA |
| 493 | GUGCGGCUCUCCUCUGGCAUG |
| 494 | GUGCGGUUCUCCUCUGGCACG |
| 495 | GUGCUCCCUUCAAACCAAUAA |
| 496 | GUGCUCCCUUCACACCAAUAA |
| 497 | GUGCUGGCGAGCUCCGGUGCCGCA |
| 498 | GUGGGUUGCGGAUAACGGUA |
| 499 | GUGUGAUGAUGUGUCAUUUAUA |
| 500 | GUGUGGCUCUCCUCUGGCAUG |
| 501 | GUGUUUGGUUUAGGGAUGAGGUGG |
| 502 | GUUCAAUAAAGCUGUGGGAA |
| 503 | GUUCGCGUCGGGUUCACCA |
| 504 | GUUCUCUACAAGCACUUCACGA |
| 505 | GUUCUCUUCAAGCACUUCACGA |
| 506 | GUUGAGCAAGUUGAAGAUGAA |
| 507 | GUUGCACGGGUUUGUAUGUUG |
| 508 | GUUUCAUCCAUGGACACCGCA |
| 509 | GUUUCCGCUCUUCAUCGAUGGCUG |
| 510 | GUUUCCUUCAAGCACUUCACAU |
| 511 | GUUUGGUGAAUCGGAAACUAUUU |
| 512 | UAAAGUCAAUAAUACCUUGAAG |
| 513 | UAACAAAGGACAACAGACUGA |
| 514 | UAACAGAGUAAUUGUACAGUG |
| 515 | UAACGCACAACACUAAGCCAU |
| 516 | UAACUAAACAUUGGUGUAGUA |
| 517 | UAACUAUUUUGAGAAGAAGUG |
| 518 | UAAGACGGAACUUACAAAGAUU |
| 519 | UAAGAUAAAGCCGUGAAUUUG |
| 520 | UAAGAUAAUGCCAUGAAUUCG |
| 521 | UAAGAUAAUGCCAUGAAUUUG |
| 522 | UAAGAUCCGGACUACAACAAAG |
| 523 | UAAGCUGCCAGCAUGAUCUUG |
| 524 | UAAGUUAAGAUUUGUGAAGAA |
| 525 | UAAUCAGUUUGGGGAGACAAA |
| 526 | UAAUCCUACCAAUAACUUCAGC |
| 527 | UAAUCUGCAUCCUGAGGUUUA |
| 528 | UAAUGAUGUGGGUACGAAUGAA |
| 529 | UAAUUGGGGAUGUUCGGUUGCU |
| 530 | UAAUUUGGUGUUUCUUCGAUC |
| 531 | UAAUUUGUAGCAAAUUGAUAGU |
| 532 | UACAUGUAUAAAAUUCUGAGGAUG |
| 533 | UACAUGUCAGUGACAAAGGCA |
| 534 | UACCAACCUUUCAUCGUUCCC |
| 535 | UACCCGAAUUUGCUUCCAUGAU |
| 536 | UACGAGCCACUGGAAACUGAA |
| 537 | UACGAGCCACUUGAAACUGAA |
| 538 | UACGCAGGAGAGAUGACGCUGU |
| 539 | UACGCAUUGAGUUUCGUUGCUU |
| 540 | UACGGAUACGGAUACGCGAUAC |
| 541 | UACUAAGUAGAGUCUAAGAGA |
| 542 | UAGAAACUUGGCUGAUGCAUUACU |
| 543 | UAGAAUGCUAUUGUAAUCCAG |
| 544 | UAGACCAUUUGUGAGAAGGGA |
| 545 | UAGACCGAUGUCAACAAACAAG |
| 546 | UAGAUGGCUGAUCUGGUGUGG |
| 547 | UAGCCAAAGAUGACUUGCCUG |
| 548 | UAGCCAAGAAUGACUUGCCGG |
| 549 | UAGCCAAGAAUGACUUGCCUA |
| 550 | UAGCCAAGAAUGGCUUGCCUA |
| 551 | UAGCCAAGGACAAACUUGCCGG |
| 552 | UAGCCAAGGAGACUGCCUAUG |
| 553 | UAGCCAAGGAUGAAUUGCCGG |
| 554 | UAGCCAAGGAUGACUUGCCGG |
| 555 | UAGCCAAGGAUGACUUGCCUA |
| 556 | UAGCCAAGGAUGACUUGCCUG |
| 557 | UAGCCAAGGAUGAUUUGCCUG |
| 558 | UAGCCCAAAGUGAGAAGAGUGGGU |
| 559 | UAGGAAUUAGUCACUCAGAUC |
| 560 | UAGGAGUGUUUGUAGGAGCGCCAC |
| 561 | UAGGAUAUGGUAAUGCUAAAA |
| 562 | UAGGCAUUUUCUCUUGGCAUG |
| 563 | UAGGGCUACUACACCAUCCAUAAG |
| 564 | UAGUCAAACUUAGAAUAGUUGGAC |
| 565 | UAGUCCACUGUGGUCUAAGGC |
| 566 | UAGUCCGGUUUUGGAUACGUG |
| 567 | UAUAGGCAUUAUUUUUUUCUUC |
| 568 | UAUCCGGCGCCGCAGGGAGG |
| 569 | UAUCGUAUCCUAGGUUGGUUU |
| 570 | UAUGAAUGGUAUAUUUUCUUG |
| 571 | UAUGAUCAUCAGAAAACAGUG |
| 572 | UAUGCGUAAGACGGAUUCGUA |
| 573 | UAUGGAUGGAGGUGUAACCCGAUG |
| 574 | UAUGGGGGGAUUGGGAAGGAA |
| 575 | UAUGGGGGGAUUGGGAAGGAAU |
| 576 | UAUGUUAACUGAUUUCAUGGAU |
| 577 | UAUUGACGCGGUUCAAUUCGA |
| 578 | UAUUGGCCUGGUUCACUCAGA |
| 579 | UAUUGGCGCGCCUCAAUCCGA |
| 580 | UAUUGGCGUGCCUCAAUCCGA |
| 581 | UAUUGGUGCGGUUCAAUCAGA |
| 582 | UAUUUGAGGAUGGAGGUAGUA |
| 583 | UAUUUUAGUUUCUAUGGUCAC |
| 584 | UCAAAAUCAGAGAAUCAACCA |
| 585 | UCAACAUGGUAUCAGAGCUGGAAG |
| 586 | UCAAGCACCAGCUCGAAGAAGC |
| 587 | UCAAGGAACGGAUUUUGUUAA |
| 588 | UCAAGGAUUAAGCUAUGAAGC |
| 589 | UCAAGUGGUGUACUCUAAAGA |
| 590 | UCAAGUUUGAUGACGAUUCCA |
| 591 | UCAAUAGAUUGGACUAUGUAU |
| 592 | UCACGGAAAACGAGGGAGCAGCCA |
| 593 | UCACUCCUCUUCUUCUUGAUG |
| 594 | UCAGAGUAUCAGCCAUGUGA |
| 595 | UCAGGUAUGAUUGACUUCAAA |
| 596 | UCAGUGCAAUCCCUUUGGAAU |
| 597 | UCAUAAGCCCACCACAUGUGG |
| 598 | UCAUCUUCAUCAUCAUCGUCA |
| 599 | UCAUGCUCCAAGAAAACCAGG |
| 600 | UCAUGGUCAGAUCCGUCAUCC |
| 601 | UCAUGUCGUAAUAGUAGUCAC |
| 602 | UCAUUCAGGAUUGAAGCCGCC |
| 603 | UCAUUGAGCGCAGCGUUGAUG |
| 604 | UCAUUUUGCGUGCAAUGAUCUG |
| 605 | UCCAAAGGGAUCGCAUUGAUC |
| 606 | UCCAAAGGGAUCGCAUUGAUCU |
| 607 | UCCAACUUGAGGCCCGAUUGA |
| 608 | UCCAAUAGGUCGAGCAUGUGC |
| 609 | UCCAAUAGGUCUAGCAUGUGC |
| 610 | UCCAAUUUGGGGAUUUGCUGAU |
| 611 | UCCACAGGCUUUCUUGAACGG |
| 612 | UCCAUAGCUUCAUCUCUGACC |
| 613 | UCCCACCGAGCAGCCGGAUCUC |
| 614 | UCCCAGAGCUUUGGCCGUCGC |
| 615 | UCCCCUCUUUAGCUUGGAGAAG |
| 616 | UCCCGACAUUAAAUUCUGGGC |
| 617 | UCCCGUGGAGGCAGCCGAUG |
| 618 | UCCGGCAAGUUGACCUUGGCU |
| 619 | UCCGGCGCCGCACUAGGCACUG |
| 620 | UCCUCCUCGGGCUCAUCGGGC |
| 621 | UCGAACUAGAAGGGCCAGGUU |
| 622 | UCGAUAAACCUCUGCAUCCA |
| 623 | UCGAUAAGCCUCUGCAUCCAG |
| 624 | UCGCUCUGAUACCAAAUUGAUG |
| 625 | UCGCUUGGUGCAGGUCGAGAA |
| 626 | UCGCUUGGUGCAGGUCGGG |
| 627 | UCGCUUGGUGCAGGUCGGGA |
| 628 | UCGGACCAGGCUUCAUCCCC |
| 629 | UCGGACCAGGCUUCAUUCC |
| 630 | UCGGACCAGGCUUCAUUCCC |
| 631 | UCGGACCAGGCUUCAUUCCCCC |
| 632 | UCGGACCAGGCUUCAUUCCCGU |
| 633 | UCGGACCAGGCUUCAUUCCCUU |
| 634 | UCGGACCAGGCUUCAUUCCUU |
| 635 | UCGGAUCAGGCUUCAUUCCUC |
| 636 | UCGGAUGCGAAACGGUGGUGU |
| 637 | UCGGCCUCGUGGAUGGACCAG |
| 638 | UCGUCGCCGGCGACCACAGC |
| 639 | UCGUGGGAUUUAUGCAGUUAA |
| 640 | UCUAAGUCUUCUAUUGAUGUU |
| 641 | UCUAGUUUGUGUUCAGCAUC |
| 642 | UCUCAGCCAGGGCAGUAACAG |
| 643 | UCUCAGUGACUAAUUUCUAGA |
| 644 | UCUCCACAGGCUUUCUUGAACU |
| 645 | UCUCCUCAGCAGCACAAGAAG |
| 646 | UCUCGCGCUUGUACGGCUUU |
| 647 | UCUCGGUUCGCGAUCCACAAG |
| 648 | UCUCUCUCUCCCUUGAAGGC |
| 649 | UCUCUCUGUUGUGAAGUCAAA |
| 650 | UCUCUUGAUUCUAGAUGAUGU |
| 651 | UCUGAUUUUCGAAGUCCCAAAA |
| 652 | UCUGCGUGAUUGAAGUCUGCAU |
| 653 | UCUGGAUCAGAGGGAGUAUA |
| 654 | UCUUCCCAAUUCCGCCCAUUCC |
| 655 | UCUUCCCUACACCUCCCAUAC |
| 656 | UCUUCUCCAAAUAGUUUAGGUU |
| 657 | UCUUGUGGCUAGAAGGGUGAG |
| 658 | UCUUUCUGCAAACGCCUUGGA |
| 659 | UGAAACCAAGUAGCUAAAUAG |
| 660 | UGAAAUGGAGGCUCGUUGUAC |
| 661 | UGAAAUUCCUGUAAAAUUCUUG |
| 662 | UGAACCUUGGGAGCGAUCUGAA |
| 663 | UGAAGAUUUGAAGAAUUUGGGA |
| 664 | UGAAGCUGCCAGCAUGAUCU |
| 665 | UGAAGCUGCCAGCAUGAUCUC |
| 666 | UGAAGCUGCCAGCAUGAUCUUA |
| 667 | UGAAGCUUCAGUUGGUUGUAU |
| 668 | UGAAGGCGACUGAUGAUUUCA |
| 669 | UGAAUCUUGAUGAUGCUGCAC |
| 670 | UGAAUCUUGAUGAUGCUGCAU |
| 671 | UGACAACUGGUAGCAGAGCAA |
| 672 | UGACAGAAGAAAGAGAGCAC |
| 673 | UGACAGAAGAGAGAGAGCACA |
| 674 | UGACAGAAGAGAGAGAGCAG |
| 675 | UGACAGAAGAGAGGGAGCAC |
| 676 | UGACAGAAGAGAGUGAGCACA |
| 677 | UGACAGAAGAGAGUGAGCACU |
| 678 | UGACAUCCAGAUAGAAGCUUUG |
| 679 | UGACAUGGGACUGCCUAAGCUA |
| 680 | UGACCCCGGUCUGCUCGCUGG |
| 681 | UGACCCCGUUCUCCUCGCCGG |
| 682 | UGACGACAACAGCUAGAAUGG |
| 683 | UGACUAGACCCGUAACAUUAC |
| 684 | UGACUGAGCCGUGCCAAUAUC |
| 685 | UGACUGAGCUUCGUUCGGUAU |
| 686 | UGACUGCAUUAACUUGAUCGU |
| 687 | UGACUUGGCAUGCUUACGUGGCAC |
| 688 | UGAGAACAAUAGGCAUGGGAGGUA |
| 689 | UGAGAAGAAGAAGAAGAAAA |
| 690 | UGAGAAUGCAAAUCCUUAGCU |
| 691 | UGAGAAUUUGGCCUCUGUCCA |
| 692 | UGAGAGAAAGCCAUGACUUAC |
| 693 | UGAGAGAAGGAAUUAGAUUCA |
| 694 | UGAGCCAAGGAUGACUUGCCG |
| 695 | UGAGCCACUGGGAUGAGGAUGAAU |
| 696 | UGAGCCAGGAUGGCUUGCCGG |
| 697 | UGAGCCAGGAUGGCUUGCCGGC |
| 698 | UGAGCCGGGAUGGCUUGCCGGCA |
| 699 | UGAGCCUCUGUGGUAGCCCUCA |
| 700 | UGAGCUGUAACACUUGAAGAC |
| 701 | UGAGGAGGAACAUAUUUACUAG |
| 702 | UGAGGGGUAGAAAUGUCAUAUCAU |
| 703 | UGAGUCGCUCUUAUCACUCAUG |
| 704 | UGAUAUCCUUGAGCUAAUACA |
| 705 | UGAUCUCUUCGUACUCUUCUUG |
| 706 | UGAUCUUGAGGCAGAAACUGAG |
| 707 | UGAUGAAUGCUGACGAUGUUG |
| 708 | UGAUGACGUGGAUGAAUUUCAAA |
| 709 | UGAUUCUCUGUGUAAGCGAAA |
| 710 | UGAUUGAGCCGCGCCAAUAUC |
| 711 | UGAUUGAGCCGUGUCAAUAUC |
| 712 | UGCACUGCCUCUUCCCUGGC |
| 713 | UGCAUCACGGCGCAUAUGUAG |
| 714 | UGCAUUUGCACCUGCACCUA |
| 715 | UGCCAAAGGAAAUUUGCCCCG |
| 716 | UGCCAAAGGAGAAUUGCCCUG |
| 717 | UGCCAAAGGAGACUUGCCCAG |
| 718 | UGCCAAAGGAGAGCUGCCCUG |
| 719 | UGCCAAAGGAGAGCUGUCCUG |
| 720 | UGCCAAAGGAGAGUUGCCCUA |
| 721 | UGCCAAAGGAGAGUUGCCCUG |
| 722 | UGCCAAAGGAGAUUUGCCCAG |
| 723 | UGCCAAAGGAGAUUUGCCCCG |
| 724 | UGCCAAAGGAGAUUUGCCCGG |
| 725 | UGCCAAAGGAGAUUUGCCCUG |
| 726 | UGCCAAAGGAGAUUUGCCUCG |
| 727 | UGCCACUCUUUUAGCUUCCGAAUC |
| 728 | UGCCGAUUUCCCCCUCGGGCG |
| 729 | UGCCUAUGUGGCACGCCACGUGAA |
| 730 | UGCCUGGCUCCCUGAAUGCCA |
| 731 | UGCCUGGCUCCCUGCAUGCCA |
| 732 | UGCCUGGCUCCCUGUAUGCCA |
| 733 | UGCCUGGCUCCCUGUAUGCCG |
| 734 | UGCGAAGUAGAGAUGCCGACU |
| 735 | UGCGAGUGUCUUCGCCUCUGA |
| 736 | UGCGAUGAUGGCCGCGCGGGUUCA |
| 737 | UGCGGAACCGUGCGGUGGCGC |
| 738 | UGCGGUGCGGGAGAAGUGC |
| 739 | UGCUAGUGAUGGUGAUUCUUCGAC |
| 740 | UGCUCAAAUACCACUCUCCU |
| 741 | UGCUCACCUCUCUUUCUGUCAGU |
| 742 | UGCUCCGGAUAUUAUGGCAUG |
| 743 | UGCUCUCUGCUCUCACUGUCAUC |
| 744 | UGCUGGGAUCGGGAAUCGAAA |
| 745 | UGGAACGGAGGAAUUUUAUAG |
| 746 | UGGAAGAAGAUGAUAGAAUUA |
| 747 | UGGAAGAAGGUGAGACUUGCA |
| 748 | UGGAAGAUGCUUUGGGAUUUAUU |
| 749 | UGGACAAGGUUAGAUUUGGUG |
| 750 | UGGACGGAGCGAUGGUGGGCG |
| 751 | UGGACUGAAGGGAGCUCCUUC |
| 752 | UGGACUGAAGGGGAGCUCCUUC |
| 753 | UGGAGAAGAUACGCAAGAAAG |
| 754 | UGGAGAAGCAGGGCACGUGUG |
| 755 | UGGAGAAGCAGGGUACGUGCA |
| 756 | UGGAGGACCUUUGAAGGUGCA |
| 757 | UGGAGGCAGCGGUUCAUCGAUC |
| 758 | UGGAUCGCUUCGUCUGAUGGU |
| 759 | UGGAUGUGACAUACUCUAGUA |
| 760 | UGGAUUCCACUGACACGUAGACGU |
| 761 | UGGAUUGGUCAAGGGAAGCGU |
| 762 | UGGCAAGUCUCCUCGGCUACC |
| 763 | UGGCAGAAGUACUGGACUUAG |
| 764 | UGGCAGAGAUUGAUCGAGGAA |
| 765 | UGGCAUAAUUCUGUCAAUUCC |
| 766 | UGGCUGAUUGACCUAAUGGCA |
| 767 | UGGCUGCUAGGCUCCUGGGUG |
| 768 | UGGCUUGGUUUAUGUACACCG |
| 769 | UGGGAAAAAUGGGCAGUUGAGU |
| 770 | UGGGACGUAUCCUAUUACUAU |
| 771 | UGGGGCUUGAUCCAAGAUAGG |
| 772 | UGGGGUUCUACAAACCGAACU |
| 773 | UGGGUGAGAGAAACGCGUAUC |
| 774 | UGGGUGGCAAACAAAGACGAC |
| 775 | UGGGUGGUGAUCAUAUAAGAU |
| 776 | UGGGUUGAGUUGAGUUGAGUUGGC |
| 777 | UGGUAAGAUUGCUUAUAAGCU |
| 778 | UGGUAGCAGUAGCGGUGGUAA |
| 779 | UGGUAUUGUUUCGGCUCAUGU |
| 780 | UGGUCUUGAGGCAGGAACUGAG |
| 781 | UGGUGAAAUUUGUAGAUUGGA |
| 782 | UGGUGAGCCUUCCUGGCUAAG |
| 783 | UGGUUUUUUUGGAGCAUGAGG |
| 784 | UGUAAAAUUCAUUCGUUCCAA |
| 785 | UGUAGGUUCCAGUGAGGGAAA |
| 786 | UGUCCCCUUCUCUGCACCACC |
| 787 | UGUCGAACCGCGGUUGUUCGA |
| 788 | UGUCGUCGUCGAUGGAGCCCAUG |
| 789 | UGUCUGCUCGAUGUCAGGUUG |
| 790 | UGUGCCAUCCCACACAUCCCGA |
| 791 | UGUGGUAUGUUGGCAAUGUAGGAA |
| 792 | UGUGUAGCCACAUUGUAAGGG |
| 793 | UGUGUUCUCAGGUCACCCCUG |
| 794 | UGUGUUCUCAGGUCACCCCUU |
| 795 | UGUGUUCUCAGGUCGCCCCCG |
| 796 | UGUUAAGGAGUGUUAACGGUG |
| 797 | UGUUCGUCCGUACACUGUUCA |
| 798 | UGUUGCGGGUAUCUUUGCCUC |
| 799 | UGUUGGCACGGUUCAAUCAAA |
| 800 | UGUUGGCAUGGUUCAAUCAAA |
| 801 | UGUUGGCCCGGCUCACUCAGA |
| 802 | UGUUGGCUCGGCUCACUCAGA |
| 803 | UGUUGGGAAAGAAAAACUCUU |
| 804 | UGUUGUUUUACCUAUUCCACC |
| 805 | UGUUUGUUGUACUCGGUCUAGU |
| 806 | UGUUUUGUGCGUGACUCUAAUU |
| 807 | UGUUUUGUGCUUGAAUCUAAUU |
| 808 | UUAAAGCUCCACCAUGAGUCCAAU |
| 809 | UUACGGCAUUAAAAGUAAAUC |
| 810 | UUAGAGUUUUCUGGAUACUUA |
| 811 | UUAGAUGACCAUCAACAAACG |
| 812 | UUAGAUUCACGCACAAACUUG |
| 813 | UUAGCUUCUUUCACCUUUCCC |
| 814 | UUAGGACUAUGGUUUGGACGA |
| 815 | UUAGGCUAAGAUUUGUGAAGA |
| 816 | UUAGGGUAGUUAACGGAAGUUA |
| 817 | UUAGUAGGACUAGAAUGGGCCAAA |
| 818 | UUAUAAGCCAUCUUACUAGUU |
| 819 | UUAUACCAAAUUAAUAGCAAA |
| 820 | UUAUAGGAGGUAUAGACGGUA |
| 821 | UUAUAGUCUGACAUCUGGAAU |
| 822 | UUAUCCGGUAUUGGAGUUGA |
| 823 | UUAUGUCUUGUUGAUCUCAAU |
| 824 | UUAUUGAGUGCAGCGUUGAUG |
| 825 | UUCAAAUGUCAGAUUAUAAAA |
| 826 | UUCAAUAAAGCUGUGGGAAG |
| 827 | UUCAGGGACUUCAAUUCAGAA |
| 828 | UUCAGUUUCCUCUAAUAUCUCA |
| 829 | UUCCAAAGGGAUCGCAUUGAU |
| 830 | UUCCAAAGGGAUCGCAUUGAUC |
| 831 | UUCCAAGCGGGCCACUUAAGCAUU |
| 832 | UUCCACAGCUUUCUUGAACU |
| 833 | UUCCACAGGCUUUCUUGAACUG |
| 834 | UUCCCAAUGCCUCCCAUGCCUA |
| 835 | UUCCCAAUGCCUUCCAUGCCUA |
| 836 | UUCCCGAUGCCUCCCAUGCCUA |
| 837 | UUCCCGAUGCCUCCCAUUCCUA |
| 838 | UUCCCGAUGCCUCCUAUUCCUA |
| 839 | UUCCUAAUGCCUCCCAUUCCUA |
| 840 | UUCCUAAUGCUUCCCAUUCCUA |
| 841 | UUCCUAGUGCCUCCCAUUCCUA |
| 842 | UUCCUAUAUGAACACUGUUGC |
| 843 | UUCCUGAUGCCUCCCAUGCCUA |
| 844 | UUCCUGAUGCCUCCCAUUCCUA |
| 845 | UUCCUGAUGCCUCCUAUUCCUA |
| 846 | UUCCUGAUGCCUCUCAUUCCUA |
| 847 | UUCCUGAUGUCUCCCAUUCCUA |
| 848 | UUCGAGGCCUAUUAAACCUCUG |
| 849 | UUCGCAGGAGAGAUAGCGCCA |
| 850 | UUCGGAUCUUUCUAGAGGCAUU |
| 851 | UUCGUUUAUUUGGACUAGAGU |
| 852 | UUCUCAAGAAGGUGCAUGAAC |
| 853 | UUCUCCGUCGCCGCCGUCCGC |
| 854 | UUCUCGAGAAGGUGCAUGAAC |
| 855 | UUCUCGAUGCCUCCCAUUCCUA |
| 856 | UUCUCUCAAGUUGCCAAACAAG |
| 857 | UUCUCUUCUUCGUGUCGCAUUU |
| 858 | UUCUUGCAUAUGUUCUUUAUC |
| 859 | UUCUUGUGCUGCUGAAGAGAC |
| 860 | UUCUUGUGGAUUCCUUGGAAA |
| 861 | UUGAACAUGGUUUAUUAGGAA |
| 862 | UUGAAGAGGACUUGGAACUUCGAU |
| 863 | UUGAAUGUGAAUGAAUCGGGC |
| 864 | UUGAAUUGAAGUGCUUGAAUU |
| 865 | UUGACAGAAGAAAGGGAGCAC |
| 866 | UUGAGAGCAACAAGACAUAAU |
| 867 | UUGAGCCGCGCCAAUAUCACU |
| 868 | UUGAGCCGCGUCAAUAUCUCA |
| 869 | UUGAGCCGCGUCAAUAUCUUA |
| 870 | UUGAGCCGUGCCAAUAUCAC |
| 871 | UUGAGCCGUGCCAAUAUCACA |
| 872 | UUGAGCCGUGCCAAUAUCACG |
| 873 | UUGAUUCCCAAUCCAAGCAAG |
| 874 | UUGCAGGAGACUAGAGACCAG |
| 875 | UUGCAUCCUCUGCACUUUGGGCCU |
| 876 | UUGCCGAUUCCACCCAUUCCU |
| 877 | UUGCUUAAAGAUUUUCUAUGU |
| 878 | UUGGACUGAAGGGAGCUCC |
| 879 | UUGGACUGAAGGGAGCUCCCA |
| 880 | UUGGACUGAAGGGAGCUCCU |
| 881 | UUGGACUGAAGGGAGCUCCUU |
| 882 | UUGGACUGAAGGGAGCUCCUUC |
| 883 | UUGGACUGAAGGGGAGCUCCUUC |
| 884 | UUGGACUGAAGGGGCCUCUU |
| 885 | UUGGACUUGAGAUUUGGUAUG |
| 886 | UUGGAUUUUUAUUUAGGACGG |
| 887 | UUGGCGGAGCGGUUGCUGUCA |
| 888 | UUGGGGACGACAUCUUUUGUUG |
| 889 | UUGGGGACGAGAUGUUUUGUUG |
| 890 | UUGGGGAUUUCCUGCCGGAGGAA |
| 891 | UUGGGGUUUCGAAAUCAAGAG |
| 892 | UUGGUGACCCAGAAGAAGUUGA |
| 893 | UUGGUGGACAAGAUCUGGGAU |
| 894 | UUGGUGUUAUGUGUAGUCUUC |
| 895 | UUGUACAAAUUUAAGUGUACG |
| 896 | UUGUAGUAACGUGAUGGUCAAUGU |
| 897 | UUGUCCCAUUCUAGUUUAGCU |
| 898 | UUGUGAUGUGAAUGAUUCAU |
| 899 | UUGUGCGAGAUCGACGGUAUA |
| 900 | UUGUGCGGUUCAAAUAGUAAC |
| 901 | UUGUUUAGGUCCCUUAGUUUC |
| 902 | UUGUUUAGGUCCCUUAGUUUCU |
| 903 | UUUAAAUCAUAUACUUUUGGU |
| 904 | UUUAACUAAUGAACCGGCACCUAU |
| 905 | UUUAGCCUCAGACCACGGUGGACU |
| 906 | UUUAGGAGAGUGGUAUUUGAG |
| 907 | UUUAGGUCGAGCUUCAUUGGA |
| 908 | UUUAGUUGACGGAAUUGUGGC |
| 909 | UUUCAAAAAUAACCUUUUGUUC |
| 910 | UUUCCCUCUCCGUGCGCGCUCG |
| 911 | UUUCCUAUGACGUCCAUUCCAA |
| 912 | UUUCCUUGGGAAGGUGGUUUC |
| 913 | UUUCGUUGUCUGUUCGACCUU |
| 914 | UUUCUUAAGUCAAACUUUUU |
| 915 | UUUGACGUGCUCGAUCUGCUC |
| 916 | UUUGAGAAGGUAUCAUGAGAU |
| 917 | UUUGAGUGUGUUUGUUAUGAA |
| 918 | UUUGAUUCCAGCUUUUGUCUC |
| 919 | UUUGCGAGUUGGCCCGCUUGC |
| 920 | UUUGCUUCCAGCUUUUGUCUC |
| 921 | UUUGGAAAUAUUUGGCUUGACU |
| 922 | UUUGGACCGAAGGGAGCCCCU |
| 923 | UUUGGACUGAAGGGAGCUCCU |
| 924 | UUUGGACUGAAGGGAGCUCUA |
| 925 | UUUGGAGUGAAGGGAGCUCUG |
| 926 | UUUGGAUCUGUUAUUUUGGUAU |
| 927 | UUUGGAUUGAAGGGAGCUCUC |
| 928 | UUUGGUUUCCUCCAAUAUCUCA |
| 929 | UUUGGUUUGAAGGGAGCUCUA |
| 930 | UUUGUUAUUUUCGCAUGCUCC |
| 931 | UUUGUUGAUUGACAUCUAUAC |
| 932 | UUUGUUGCAAUUUGGACUACC |
| 933 | UUUGUUUUCCUCCAAUAUCUCA |
| 934 | UUUGUUUUCCUCUAAUAUCUCA |
| 935 | UUUUACUGCUACUUGUGUUCC |
| 936 | UUUUCUUCUACUUCUUGCACA |
| 937 | UUUUCUUGGCCCAUCCACUUC |
| 938 | UUUUGGAACGGAGUGAGUAUU |
| 939 | UUUUGGACUGAAGGGAGCUCC |
| 940 | UUUUGUAUGUUGAAGGUGUAU |
| 941 | UUUUGUGUCGUGAAGCUUUUG |
| 942 | UUUUUCCUCAAAUUUAUCCAA |
