# Supplementary material for: Prediction of plant-derived xenomiRs from plant miRNA sequences using random forest and one-dimensional convolutional neural network models
Source: BMC Genomics. 2018 Nov 26;19:839. doi: 10.1186/s12864-018-5227-3 (PMC6258294; doi:10.1186/s12864-018-5227-3)
Supplement: Supplementary file 3 — Table S3. Feature list. (DOCX 14 kb) [file 12864_2018_5227_MOESM3_ESM.docx]

| **Region** | **Feature category** | **Number of features** |
| --- | --- | --- |
| Full sequence | Length | 1 |
|  | Nucleotide position | 24 |
|  | Motif frequency (1~3 nt) | 84 |
| Seed region | Motif frequency (1~2 nt) | 20 |
