# Supplementary material for: Prediction of plant-derived xenomiRs from plant miRNA sequences using random forest and one-dimensional convolutional neural network models
Source: BMC Genomics. 2018 Nov 26;19:839. doi: 10.1186/s12864-018-5227-3 (PMC6258294; doi:10.1186/s12864-018-5227-3)
Supplement: Supplementary file 5 — Table S5. The top 10% most important features evaluated by RF model using mean decrease accuracy (left) and mean decrease Gini (right), respectively. (DOCX 14 kb) [file 12864_2018_5227_MOESM5_ESM.docx]

| Rank | Mean Decrease Accuracy | Mean Decrease Gini |
| --- | --- | --- |
| 1 | G | CAG |
| 2 | U | C |
| 3 | A | CA |
| 4 | C | UAU |
| 5 | GG | U |
| 6 | UG | G |
| 7 | CA | A |
| 8 | UC | UA |
| 9 | CAG | AU |
| 10 | UU | UC |
