# Supplementary material for: Prediction of plant-derived xenomiRs from plant miRNA sequences using random forest and one-dimensional convolutional neural network models
Source: BMC Genomics. 2018 Nov 26;19:839. doi: 10.1186/s12864-018-5227-3 (PMC6258294; doi:10.1186/s12864-018-5227-3)
Supplement: Supplementary file 7 — Table S7. The 241 potential miRNAs predicted by both RF model and 1D-CNN model. (DOCX 23 kb) [file 12864_2018_5227_MOESM7_ESM.docx]

| miR408a | AAGACUGGGAACAGGCAGAGCA |
| --- | --- |
| miR2592b-5p | ACAACAGGACUCAAGCAUUUC |
| miR7829 | ACACAGAAACUCCAAGCCCAC |
| miR402-3p | ACAGAGUUCCGUAGACCACGG |
| miR408b | ACAGGGAAGAGGUAGAGCAUG |
| miR408a-5p | ACAGGGACGAGGCAGCGCAUG |
| miR4403 | ACGGACACCGAACACGACACGGAC |
| miR408b-5p | ACGGGGACGAGACAGAGCAUG |
| miR8011b-5p | ACUCAUUUUUGUCUCACAAAAA |
| miR408b | ACUGGGAACAGGCAGAGCAUGA |
| miR172b | AGAAUCAUGAUGAUGCUGCAU |
| miR172f | AGAAUCCUGAUGAUGCUGCAC |
| miR172d | AGAAUCCUGAUGAUGCUGCAG |
| miR172d | AGAAUCCUGAUGAUGCUGCAU |
| miR172b | AGAAUCUUGAUGAUGCGGCAA |
| miR172c-3p | AGAAUCUUGAUGAUGCUGC |
| miR172a-3p | AGAAUCUUGAUGAUGCUGCGU |
| miR1023a-3p | AGAGAAUUGGAGAGAGUGCAU |
| miR6439b | AGCAGAAGCCAUCACUAGCGC |
| miR1172.1 | AGGAUUGCAGCAGCAACGGGGC |
| miR1520d | AUCAGAACAUGACACGUGACAA |
| miR319c | AUCCAACGAAGCAGGAGCUGA |
| miR9757 | CAACCCUCCUCAGUUAGAUCUC |
| miR853-3p | CAACCGGAAAGGGGAGCAGCA |
| miR5497 | CAGAAUAUCUGGGACGAGCAU |
| miR1447 | CAGAAUUGCAGUGCCUUGAUU |
| miR4407 | CAGAGGAAGCAGCACUUGUACC |
| miR167f-3p | CAGAUCAUGCUGCAGCUUCAU |
| miR169d | CAGCCAAGAAUGAUUUGCCGG |
| miR7760-5p | CAGCGGACAGAAUGGAGCAAGCAG |
| miR408-5p | CAGGGAUGGAGCAGAGCAAGG |
| miR408-5p | CAGGGAUGGAGCAGAGCAUGG |
| miR408a-5p | CAGGGGAACAGGCAGAGCAUG |
| miR947 | CAUCGGAAUCUGUUACUGUUUC |
| miR4397-5p | CAUCGUUGACGCUGACUGUACG |
| miR7527 | CAUGGCGUGCAAAACCCCACGC |
| miR5561-5p | CAUUUGGAGAGACAUAGACAA |
| miR6145a | CAUUUUCACAUGUAGCACUGAC |
| miR1148.1 | CCAACGUGCAGGGGGACAUGG |
| miR6199 | CCACAGAAUUCUCACAGUGAUGG |
| miR5742 | CCACAUCAAUGGUCGUUGGAU |
| miR2625 | CCAUCGUGCCACGUUACGAUCC |
| miR397 | CCAUUGAGUGCAGCGUUGAUG |
| miR168a-3p | CCCGCCUUGUAUCAAGUGAAU |
| miR168b-3p | CCCGUCUUGUAUCAACUGAAU |
| miR8130-3p | CCCUUCCAGUAAGGCACCCCC |
| miR166c | CCGGACCAGGCUUCAUCCCAG |
| miR397a | CCGUUGAGUGCAGCGUUGAUG |
| miR482d | CCUUUCCAACGCCUCCCAUGCC |
| miR5181e | CGACACUUACUGUGGCUCGGA |
| miR6214 | CGACGACGACGAGCACGACA |
| miR6186 | CGAGGAAGGCGCUGAGAGAGA |
| miR408-5p | CGGGGAACAGGCAGAGCAUGG |
| miR5636 | CGUAGUUGCAGAGCUUGACGG |
| miR403-3p | CUAGAUUCACGCACAAGCUCG |
| miR533c | CUCACAGUCUGCACAGCUCUC |
| miR533e | CUCACAGUCUGCAUGGCUCUC |
| miR1432 | CUCAGGAAAGAUGACACCGAC |
| miR396d | CUCCACGGCUUUCUUGAACUU |
| miR395 | CUGAAGCGUUUGGGGGAAGG |
| miR395a | CUGAAGUGUUUGGGGGAACUCC |
| miR395b-3p | CUGAAGUGUUUGGGGGGACCC |
| miR395e | CUGAAGUGUUUGGGGGGACUC |
| miR395c-3p | CUGAAGUGUUUGGGGGGACUU |
| miR156f-5p | CUGACAGAAGAGAGUGAGCA |
| miR156r | CUGACAGAAGAUAGAGAGCAU |
| miR408e | CUGCACUGACUCUUCCCUGGC |
| miR408a | CUGCACUGCAUCUUCCCUGUGC |
| miR408-5p | CUGGGAACAGGCAGAGCAUGA |
| miR1219b | CUUCCUGCCUCUCACUAGCUU |
| miR159c | GAAUUCCUUCUCCUCUCCUUU |
| miR167c-3p | GAUCAUGACUGACAGCCUCAUU |
| miR5564b | GCAAUUCGUCGAACAGCUUGA |
| miR160e-3p | GCAUGAGGGGAGUCGAGCAGG |
| miR160b-3p | GCGUAUGAGGAGCCAUGCAUA |
| miR156b-3p | GCUCACUUCUCUCUCUGUCACC |
| miR167 | GGAAGCUGCCAGCAUGAUCCU |
| miR172a | GGAAUCUUGAUGAUGCUGCA |
| miR172 | GGAAUCUUGAUGAUGCUGCAG |
| miR172e | GGAAUCUUGAUGAUGCUGCAGCAG |
| miR165a-5p | GGAAUGUUGUCUGGAUCGAGG |
| miR166k-5p | GGAUUGUUGUCUGGCUCGGGG |
| miR166n-5p | GGAUUGUUGUCUGGCUCGGUG |
| miR7841 | GGGGGUUGCUGUCAAGCAUAA |
| miR5168-5p | GGGUUGUUGUCUGGUUCAAGG |
| miR396g-3p | GUUCAAGAAAGCUGUGGAAGA |
| miR396a-3p | GUUCAAGAAAGCUGUGGGAAA |
| miR167b-5p | UAAAGCUGCCAGCAUGAUCUGG |
| miR169l-5p | UAGCCAGGGAUGAUUUGCCUG |
| miR1155 | UAGUCCUGCACGAGGAAGGAGC |
| miR167e | UCAAGCUGCCAGCAUGAUCUA |
| miR3623-5p | UCACAAGUUCAUCCAAGCACCA |
| miR8585 | UCACAGGAGAGAUGAUACUGGU |
| miR3433-3p | UCACAGUUCUUGAUUACCCAC |
| miR1115-3p | UCACCAAAGUGCCUGAGCUCA |
| miR6437a | UCACGGACGGCGGCUCCAAGCA |
| miR167c | UCAGAUGAAGCUGCCAGCAUGA |
| miR8602 | UCAGGAGAGAUGAUGCCGGCC |
| miR397 | UCAUUGAGUGCAGCGUUGACG |
| miR397a | UCAUUGAGUGCAGCGUUGAUGU |
| miR397b | UCAUUGAGUGCAGCGUUGGUG |
| miR397b | UCAUUGAGUGCAUCGUUGAUG |
| miR6473 | UCCACAAUCCCAUCAAGACUU |
| miR396a | UCCACAGGCUUUCUUGAACAU |
| miR8035 | UCCAUCUUCAAUAUCACUUUCU |
| miR396d | UCCCACAGCUUUAUUGAACUG |
| miR396 | UCCCACGGCUUUCUUGAACUU |
| miR7809 | UCCCAUUGCAUCAGCGGACACA |
| miR5300 | UCCCCAGUCCAGGCAUUCCAAC |
| miR906-3p | UCCGAUAAAGCUUCCCCCUGC |
| miR482d | UCCUCCCUACUCCUCCCAUU |
| miR7121h | UCCUCUUGGUGAUCGCCCUGC |
| miR162 | UCGAUAAACCUCUGCAUCCGG |
| miR162a | UCGAUAAACCUGUGCAUCCAG |
| miR5225c | UCGCAGGAGAGAUGACACCUUC |
| miR3627a | UCGCAGGAGAGAUGGCACUA |
| miR3627-5p | UCGCAGGAGAGAUGGCACUGUC |
| miR3627-5p | UCGCAGGAGAGAUGGCACUUAG |
| miR166m | UCGGACCAGGCAUCAUUCCUU |
| miR166p | UCGGACCAGGCUCCAUUCCUU |
| miR166j-3p | UCGGACCAGGCUUCAAUCCCU |
| miR166b | UCGGACCAGGCUUCAUUCCUA |
| miR166i | UCGGACCAGGCUUCAUUCUC |
| miR166d | UCGGGCCAGGCUUCAUCCCCC |
| miR919.2 | UCUCAGGAGGACAUCGCCACU |
| miR530 | UCUGCAUUUGCACCUGCACCU |
| miR482a-3p | UCUUCCCUAUGCCUCCCAUUCC |
| miR482c | UCUUCCCUAUUCCUCCCAUU |
| miR482-3p | UCUUCCUUGUUCCUCCCAUU |
| miR482b | UCUUGCCCACCCCUCCCAUUCC |
| miR482b | UCUUGCCUACUCCACCCAUGCC |
| miR482a.2 | UCUUGCCUACUCCUCCCAUU |
| miR482 | UCUUUCCAAUUCCUCCCAUUCC |
| miR482c-3p | UCUUUCCGAGUCCUCCCAUACC |
| miR482c | UCUUUCCUAACCCUCCCAUUCC |
| miR482f | UCUUUCCUACUCCACCCAUUCC |
| miR482e-3p | UCUUUCCUACUCCUCCCAUACC |
| miR482b | UCUUUCCUAUCCCUCCCAUUCC |
| miR3701 | UGAACAAUGCCCACCCUUCAUC |
| miR5740 | UGAACAGAAAGAACAUUUGGC |
| miR6187 | UGAACAGGUUCGGCGACCUCA |
| miR167b | UGAAGCUGACAGCAUGAUCUA |
| miR167h-5p | UGAAGCUGCCAACAUGAUCUG |
| miR167a | UGAAGCUGCCAGCAUGAUCUAA |
| miR167c-5p | UGAAGCUGCCAGCAUGAUCUGC |
| miR167k | UGAAGCUGCCAGCCUGAUCUUA |
| miR9661-5p | UGAAGUAGAGCAGGGACCUCA |
| miR395p-3p | UGAAGUGUUUGGAGGAACUC |
| miR395d | UGAAGUGUUUGGGGGAACUUU |
| miR172f | UGAAUCUUGAUGAUGCCGCAC |
| miR9674c-5p | UGAAUUUUUCCAUAGCAUCAG |
| miR535 | UGACAACGAGAGAGAGCACGCG |
| miR535b | UGACAACGAGAGAGAGCACGG |
| miR535a | UGACAACGAGAGAGAGCACGU |
| miR535 | UGACAACGAUAGAGAGCACGC |
| miR1160.2 | UGACAAGGAAGCAGAGCGGAU |
| miR535b | UGACAAGGAGAGAGAGCACGC |
| miR535 | UGACAAUGAGAGAGAGCACAC |
| miR156h | UGACAGAAGAGAGAGAGCAU |
| miR156c | UGACAGAAGAGAGGGAGCA |
| miR156b | UGACAGAAGAGAGUGAGCAUA |
| miR156a | UGACAGAAGAGAGUGAGUAC |
| miR156j | UGACAGAAGAGGGUGAGCAC |
| miR6147 | UGACAUCUUCAAAACCCACUA |
| miR2633 | UGACAUUUUGCUCCAGAUUCA |
| miR7725a-5p | UGACGAGAUCACAUCGUUUGCACA |
| miR3522b | UGAGACCAAAUGAGCAGCUGAC |
| miR8123-5p | UGAGCAAUGGCACACAGCCCU |
| miR156e-5p | UGAUAGAAGAGAGUGAGCAC |
| miR408a-3p | UGCACAGCCUCUUCCCUGGUU |
| miR408-3p | UGCACUGCCUCUUCCCUGCC |
| miR4376 | UGCAGGAGAGAUGACGCCCAUC |
| miR1144a.2 | UGGAACCGGGCACGCAGGAG |
| miR3954 | UGGACAGAGAAAUCACGGUCA |
| miR6207 | UGGACGACCUGGGCGCCGACG |
| miR164c | UGGAGAAGCAGGACACGUGAG |
| miR164a | UGGAGAAGCAGGGCACAUGCC |
| miR164d | UGGAGAAGCAGGGCACAUGCU |
| miR164d | UGGAGAAGCAGGGCACGUGC |
| miR164e-5p | UGGAGAAGCAGGGCACGUGCAA |
| miR164b | UGGAGAAGCAGGGCACUUGCU |
| miR164b | UGGAGAGGCAGGGCACAUGCU |
| miR1100 | UGUCACGGACAGAACCCCACUC |
| miR156c | UGUCAGAAGAGAGUGAGCAC |
| miR1509b | UUAAUCUAGGAAAUUACACUCG |
| miR482b-3p | UUACCAAUACCUCUCAUGCCAA |
| miR2089-5p | UUACCUAUUCCACCAAUUCCAU |
| miR827 | UUAGAUGACCAUCAACGAAAA |
| miR827 | UUAGAUGACCAUCAACGAACA |
| miR7122b | UUAUACAGAGAAAUCACGGUCG |
| miR9472-3p | UUCACAAUCUCUGCUGAAAAA |
| miR7505 | UUCAGAAACCAUCCCUUCCUU |
| miR1156.2 | UUCAGCUGGAGCUUCAGGCAC |
| miR909.3 | UUCAGGGUCAAGUUUGCAUGC |
| miR396a | UUCCACAGCUUUCUUGAACAG |
| miR396a | UUCCACAGCUUUCUUGAACGU |
| miR396a | UUCCACAGCUUUCUUGAACUA |
| miR396e | UUCCACAGCUUUCUUGAACUGU |
| miR396 | UUCCACGGCUUUCUUGAACC |
| miR2950-5p | UUCCAUCUCUUGCACACUGGA |
| miR482c-3p | UUCCCAAGCCCGCCCAUUCCAA |
| miR482a-3p | UUCCCAAGCCCGCCCAUUCCUA |
| miR6118-3p | UUCCGAGGCCACCCAUUCCAAC |
| miR3443-5p | UUCUAAUCCCGCCAUGCACCG |
| miR5050 | UUGAACGACCUCACCAUGUCG |
| miR395k | UUGAAGCGUUUGGGGGAACUC |
| miR395g | UUGAAGUGUUUGGGGGAACUC |
| miR395a | UUGAAGUGUUUGGGGGGACUC |
| miR156c | UUGACAGAAGAAAGAGAGCAC |
| miR156d | UUGACAGAAGACAGGGAGCAC |
| miR156f | UUGACAGAAGAGAGAGAGCACA |
| miR156 | UUGACAGAAGAUAGAGAGC |
| miR156l | UUGACAGAAGAUGGAGAGCAC |
| miR162 | UUGAUAAACCUCUGCAUCCAG |
| miR6149-5p | UUGCAACACACCUGAAUCGUC |
| miR482d-3p | UUGCCGACCCCACCCAUGCCAA |
| miR9660-5p | UUGCGAGCAACGGAUGAAUC |
| miR168a | UUGCUUGGUGCUGGUCGGGAA |
| miR319c | UUGGACUGAAAGGAGCUCCU |
| miR319b | UUGGACUGAAGGGUGCUCCC |
| miR319-3p | UUGGACUGAAGGGUUCCCUUC |
| miR8152 | UUGGACUGCUAGGUGGCCCAU |
| miR159b | UUGGAUUGAAGAGAGCUCCC |
| miR159a | UUGGAUUGAAGGGAGCUCCA |
| miR4229 | UUGGCAGGGAGGUCCUCCACAC |
| miR394a | UUGGCAUUCUGUCCACCUCCAU |
| miR9471a-3p | UUGGCUGAGUGAGCAUCACGG |
| miR9471b-3p | UUGGCUGAGUGAGCAUCACUG |
| miR319i | UUGGGCUGAAGGGAGCUCCC |
| miR3627-5p | UUGUCGCAGGAGAGACGGCACU |
| miR1510a-5p | UUGUCUUACCCAUUCCUCCCA |
| miR7777-3p.1 | UUGUUCCACCCAACAGAAGAU |
| miR5041-5p | UUUCAUCUUCAACUUGCUCAA |
| miR2118 | UUUCCUAUUCCACCCAUCCCAU |
| miR482d-3p | UUUCCUAUUCCACCCAUGCCAA |
| miR1219d | UUUCCUGCCUCUCACUAGCUU |
| miR397 | UUUGAGUGCAGCGUUGAUGA |
| miR159e | UUUGGAUUGAAAGGAGCUCUU |
| miR6471 | UUUGGGAUCAUCAGGACAGCC |
| miR5179 | UUUUGCUCAAGACCGCGCAAC |
| miR472-3p | UUUUUCCUACUCCGCCCAUACC |
