# Supplementary figures and images for: Prediction of plant-derived xenomiRs from plant miRNA sequences using random forest and one-dimensional convolutional neural network models
Source: BMC Genomics. 2018 Nov 26;19:839. doi: 10.1186/s12864-018-5227-3 (PMC6258294; doi:10.1186/s12864-018-5227-3)

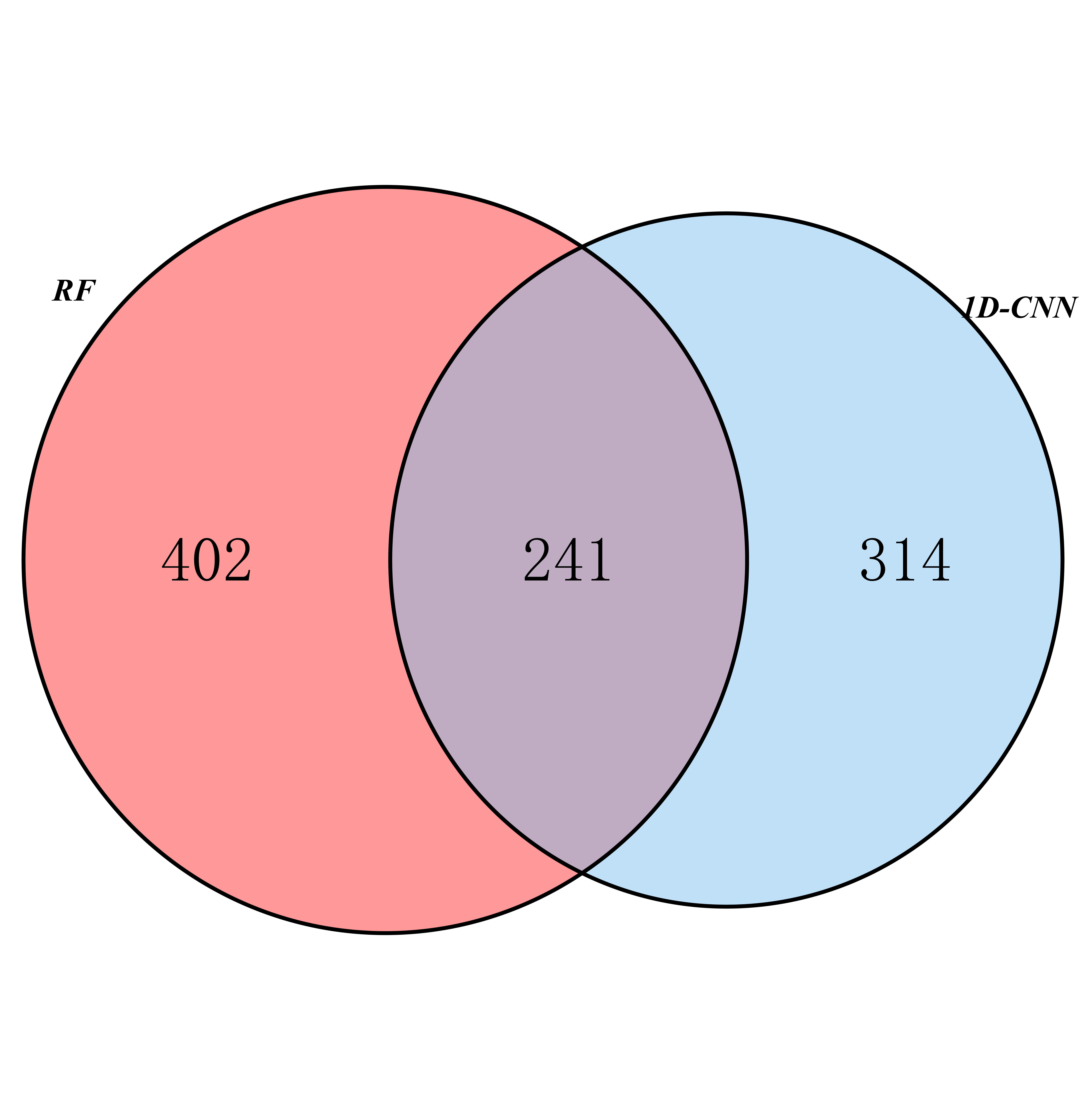

Supplement: Supplementary file 9 — Table S8. The 2194 unique target genes predicted by both miRanda and RNAhybrid. The genes were encoded in entrez ID. (TIF 843 kb) [file 12864_2018_5227_MOESM9_ESM.tif]
