# Supplementary material for: Prediction of plant-derived xenomiRs from plant miRNA sequences using random forest and one-dimensional convolutional neural network models
Source: BMC Genomics. 2018 Nov 26;19:839. doi: 10.1186/s12864-018-5227-3 (PMC6258294; doi:10.1186/s12864-018-5227-3)
Supplement: Supplementary file 11 — Table S9. Accuracy measurement. (DOCX 22 kb) [file 12864_2018_5227_MOESM11_ESM.docx]

| Metric | Formulas |
| --- | --- |
| ACC | $\frac{TP+TN}{TP+FP+FN+TN}$ |
| SN | $\frac{TP}{TP+FN}$ |
| SP | $\frac{TN}{TN+FP}$ |
| MCC | $\frac{TP\times TN-FP\times FN}{\sqrt{(TP+FN)(TP+FP)(TN+FP)(TN+FN)}}$ |

TP, FP, TN and FN are the numbers of true positives, false positives, true negatives, and false negatives, respectively. ACC, SN, SP and MCC indicate accuracy, sensitivity, specificity and Mathews correlation coefficient.
